# Smoking induces DNA methylation changes in Multiple Sclerosis patients with exposure-response relationship

**DOI:** 10.1038/s41598-017-14788-w

**Published:** 2017-11-06

**Authors:** Francesco Marabita, Malin Almgren, Louise K. Sjöholm, Lara Kular, Yun Liu, Tojo James, Nimrod B. Kiss, Andrew P. Feinberg, Tomas Olsson, Ingrid Kockum, Lars Alfredsson, Tomas J. Ekström, Maja Jagodic

**Affiliations:** 1 Department of Clinical Neuroscience, Center for Molecular Medicine, Karolinska Institutet, Karolinska University Hospital, Stockholm, Sweden; 2 Department of Biochemistry and Molecular Biology, The Ministry of Education Key Laboratory of Metabolism and Molecular Medicine, School of Basic Medical Sciences, Fudan University, Shanghai, China; 3 State Key Laboratory of Medical Neurobiology, Fudan University, Shanghai, China; 4 Center for Epigenetics and Departments of Medicine, Biomedical Engineering, and Mental Health, Johns Hopkins University, Baltimore, MD USA; 5 Institute of Environmental Medicine, Karolinska Institutet, Stockholm, Sweden

## Abstract

Cigarette smoking is an established environmental risk factor for Multiple Sclerosis (MS), a chronic inflammatory and neurodegenerative disease, although a mechanistic basis remains largely unknown. We aimed at investigating how smoking affects blood DNA methylation in MS patients, by assaying genome-wide DNA methylation and comparing smokers, former smokers and never smokers in two Swedish cohorts, differing for known MS risk factors. Smoking affects DNA methylation genome-wide significantly, an exposure-response relationship exists and the time since smoking cessation affects methylation levels. The results also show that the changes were larger in the cohort bearing the major genetic risk factors for MS (female sex and HLA risk haplotypes). Furthermore, CpG sites mapping to genes with known genetic or functional role in the disease are differentially methylated by smoking. Modeling of the methylation levels for a CpG site in the *AHRR* gene indicates that MS modifies the effect of smoking on methylation changes, by significantly interacting with the effect of smoking load. Alongside, we report that the gene expression of *AHRR* increased in MS patients after smoking. Our results suggest that epigenetic modifications may reveal the link between a modifiable risk factor and the pathogenetic mechanisms.

## Introduction

Multiple Sclerosis (MS), a leading cause of neurological disability in young adults, is a chronic inflammatory disease characterized by autoimmune destruction of myelin sheaths and subsequent neuronal death. Although the cause of MS remains unknown, vast epidemiological data establish MS as a complex disease influenced by genetic and environmental factors. Genome-wide and custom-designed array association studies have identified a large number of genetic variations that predispose to MS^1–3^. However, while non-MHC loci have a modest effect (Odds Ratio, OR < 1.3), only associations with HLA genes yield higher OR, with HLA-DRB1*15:01 exerting the strongest influence (OR > 3). The epidemiological data, together with a modest concordance rate of MS in monozygotic twins^4,5^, suggest nonetheless an important role of environmental factors that act at the population level. One of the most established environmental risk factors for MS is cigarette smoking. Both active smoking and exposure to passive smoke have repeatedly been associated with an increased risk of developing MS, disease progression and clinical disability^6–9^. In contrast to other MS risk factors, smoking increases MS risk regardless of the age of exposure, with both duration and intensity of smoking contributing independently to the increased risk of MS^7,8^. Interestingly, the effect of smoking on the risk of MS persists up to five years after smoking cessation and is reversed a decade after cessation. However, while it is difficult to associate a heterogeneous compound such as cigarette smoke with specific mechanisms of action, it appears that lung irritation due to burned tobacco products alters MS risk, likely causing local oxidative stress and pro-inflammatory response contrary to the systemic nicotine use such as snuff^7^. Notably, there is an important gene-environment interaction between cigarette smoking (and passive smoking to a lesser extent) and the established HLA alleles for MS^10^. Indeed, in Scandinavians who are non-smokers, carriage of the major risk allele HLA-DRB1*15:01 and absence of the protective *HLA-A2* variant confer an OR~5, while in smokers this OR increases to ~14^10^.

Epigenetic mechanisms, such as DNA methylation, integrate both internal and external cues and may lead to stable but reversible changes in gene expression. DNA methylation in blood from smokers has been extensively studied and has yielded several well-replicated loci where methylation levels associate with smoking intensity and time from cessation^11,12^. More recently, DNA methylation has been suggested to play a crucial role in gene-smoking interaction in immune diseases such as rheumatoid arthritis^13^. However, while studies investigating epigenetic mechanisms in MS exist^14–16^, our understanding of the contribution of DNA methylation is still incomplete, especially when genetic, epigenetic and environmental determinants are incorporated in the study design, with the goal of understanding their interaction and how they collectively affect susceptibility to disease^17^.

Therefore, the aim of this study was to examine the effect of smoking on DNA methylation in blood cells from MS patients, in order to better delineate the role of epigenetic changes in relation to one important risk factor and disease modifier, and eventually contribute to the understanding of the pathogenetic mechanisms.

## Results

### The effect of smoking on blood DNA methylation in MS patients

We evaluated the effect of cigarette smoking on DNA methylation using two independent cohorts obtained from a larger EIMS (Epidemiological Investigation of Multiple Sclerosis) project, a population-based case–control study of MS in Sweden. The “Selected cohort” (S, 50 MS patients, Supplementary Table 1) included only Swedish female subjects selected for being carriers of the HLA-DRB1*15:01 allele (DR15+/+ or DR15+/−) and non-carriers of the HLA-A*02 allele (A2−/−). HLA-DRB1*15:01 and HLA-A*02 are the major risk and protective variants for MS, respectively, and both significantly interact with smoking^10^. On the contrary, the “Broad cohort” (B) included Swedish subjects without any further selection criteria, which allowed us to include also healthy controls in the experimental design. This cohort was not restricted to any sex or genetic risk carriers and included 132 MS patients and 135 controls (Supplementary Table 1). CpG methylation from whole blood was profiled genome-wide using the Illumina HumanMethylation450k BeadChips and, in light of the above differences, the cohorts were first analyzed separately to identify Differentially Methylated Positions (DMPs) with a linear model that corrected for potential confounding factors. Next, we performed a meta-analysis to further identify robust DMPs associated with smoking in MS. The grouping into smoking categories was guided by previous epidemiological observations regarding the influence of smoking on the risk of MS. Since it has previously been shown that the increased risk for MS associated with smoking remained up to 5 years after smoking cessation^7^, the samples were classified either as W5Y (Within 5 Years), B5Y (5 Years and Beyond) or NS (never-smokers), by considering the time of sampling relative to the last smoking event, i.e. <5 years or ≥5 years for W5Y and B5Y, respectively.

**Figure 1:**
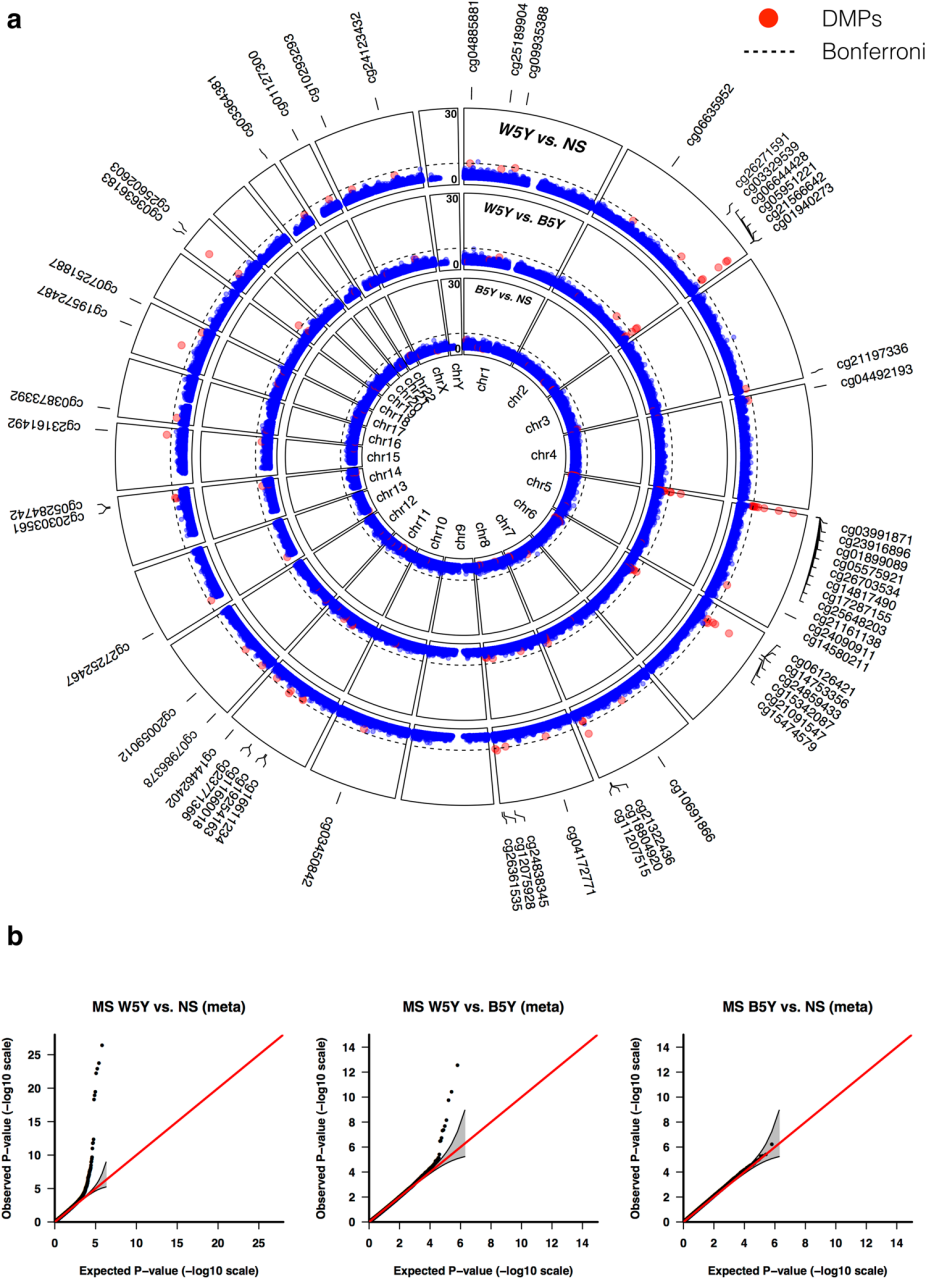
Smoking induces DNA methylation changes that are attenuated five years after smoking cessation. (**a**) A circular Manhattan plot is shown for the −log_10_P-values obtained from the meta-analysis of the S and B cohorts, for the comparisons W5Y *vs*. NS, W5Y *vs*. B5Y and B5Y *vs*. NS (outmost to innermost circle). A dashed line indicates the genome-wide significant level (Bonferroni, 1.14 × 10^−07^) and a red dot marks the 58 DMPs selected as indicated in the main text. (**b**) P-P plot of the expected *vs*. observed −log_10_P-values for the comparisons W5Y *vs*. NS, W5Y *vs*. B5Y and B5Y *vs*. NS respectively. The 0.95 confidence interval is indicated by a grey shaded area and is calculated under the assumption of the P-values being drawn independently from a uniform [0, 1] distribution.

With MS cases, five and nine positions exceeded the Bonferroni genome-wide significance threshold (1.14 × 10^−07^) in the S and B cohort, respectively, when comparing W5Y *vs*. NS (Supplementary Table 2). However, after meta-analysis, 28, 7 and 0 positions reached the genome-wide significance threshold for W5Y *vs*. NS, W5Y *vs*. B5Y and B5Y *vs*. NS, respectively (Fig. 1a). Selected CpGs were technically validated using bisulfite pyrosequencing (Supplementary Figure 1). The analysis of P-P plots (Fig. 1b) showed that the effect appeared stronger when comparing W5Y *vs*. NS, while only a minor effect remained when comparing W5Y *vs*. B5Y, and no significant effect was detected for the contrast B5Y *vs*. NS. Most importantly, no evidence of systematic bias in our analytical approach was observed.

In addition to the models presented above, which accounted for unknown variation using Surrogate Variables (SVs), we also performed explicit cell-type-adjustment, by estimating the blood cell type proportions using a reference-based method^18^. We detected no major significant differences in the estimated relative fractions between the different smoking categories (Supplementary Figure 2). Moreover, the −log_10_(P_val_) for the SV- and cell-type-adjusted models were correlated and the top cohort-specific DMPs were preserved after cell-type adjustment. We therefore selected the SV-adjusted primary models for meta-analysis and concluded that the effect of smoking on DNA methylation did not appear to be confounded by difference in cell type compositions between smokers and non-smokers. Similarly, the explicit correction for the risk haplotype as binary covariate in the B cohort (DR15+ and A2−/−) did not substantially alter the association results in this cohort (Supplementary Figure 3).

To further exclude sources of heterogeneity between the cohorts, which would compromise the legitimacy of our meta-analysis, we considered the overlap of the ranked DMP lists of the cohort-specific analyses (correspondence at the top^19^), hypothesizing that DMPs with a similar effect in the two cohorts should be top-ranked in both the corresponding primary analyses and therefore suitable for meta-analysis. Indeed, we verified the overlap between the two analyzed cohorts, which was substantial for the top-ranked DMPs for W5Y *vs*. NS, partial for W5Y *vs*. B5Y and not different from random for B5Y *vs*. NS (Supplementary Figure 4). This indicates that a number of DMPs are similarly modulated by the smoking behavior in the two cohorts, with an effect size that is attenuated with the increasing time since smoking cessation.

**Figure 2:**
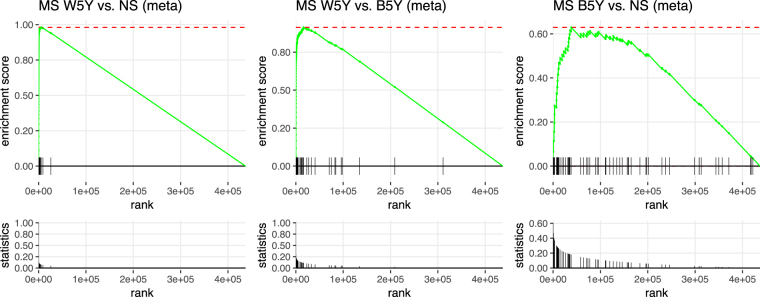
DNA methylation changes are enriched for known sites affected by smoking. A GSEA approach was used to demonstrate that enrichment exists for known smoking-affected CpGs. The green line on the top panels shows the Enrichment Score. Vertical ticks mark the location of the 62 CpGs from Gao *et al*.^11^ within the list of the probes ranked by decreasing significance, and the bottom panels show their corresponding normalized −log_10_P-values (for the comparisons W5Y *vs*. NS, W5Y *vs*. B5Y and B5Y *vs*. NS, respectively).

**Figure 3:**
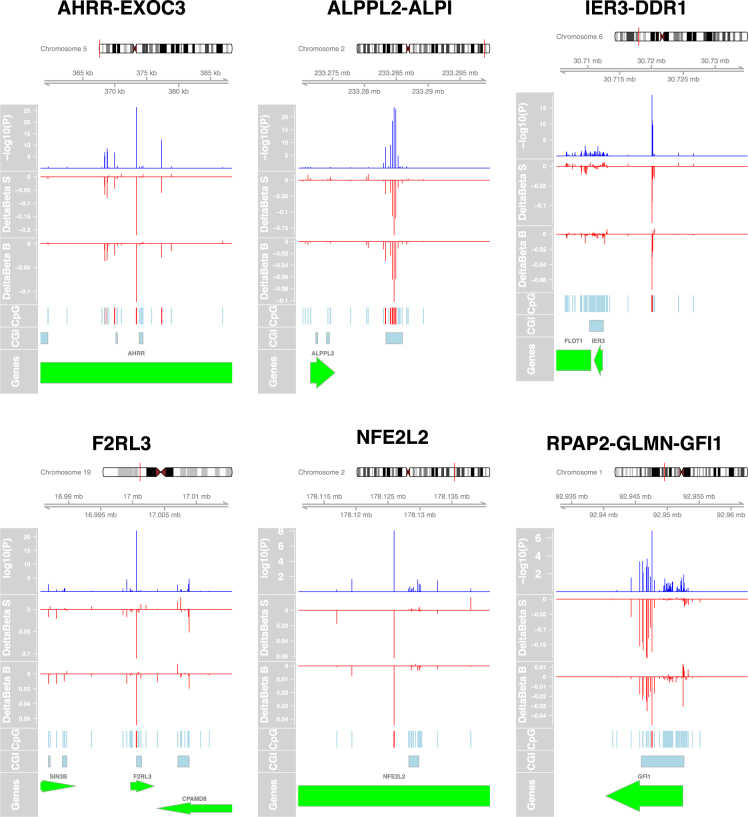
Genomic localization of selected DMPs. For six selected DMPs, the panels show: the meta-analysis P-value, the effect size (Δβ) for the S and B cohorts, the location of all 450k probes, the CpG islands and the corresponding genes in the region. The plots are centered on the most significant DMPs and extended ± 15000 bp.

Nevertheless, with a relatively small sample size and under the hypothesis of spatially correlated CpG sites, the Bonferroni adjustment appears exceedingly conservative. Hence, we examined the validity of our results by comparing our ranked lists with CpG sets obtained from the literature and for which the effect of smoking has been extensively validated. We retrieved the set of 62 CpG sites from Gao *et al*.^11^, which included only known associations reported by at least three studies, and performed enrichment analysis on the probes from this study ranked by decreasing significance (Fig. 2). We observed a sizeable enrichment of the known loci at the top of the list for the contrast W5Y *vs*. NS (Enrichment Score = 0.98, P < 10^−4^), confirming that despite the small sample size, our results are appropriate to detect the effect of smoking on DNA methylation. Therefore, we obtained a final list of 58 DMPs (Table 1), considering only the positions having: (i) an FDR < 0.05 in the main comparison W5Y *vs*. NS and (ii) a corresponding change in the beta value scale (Δβ) larger than the 98th percentile of all the Δβ values. The majority of the 58 selected DMPs (84%) were found hypomethylated after smoking, with a maximum absolute average Δβ of 22% and 12% in the S and B cohort, respectively. Besides the manufacturer’s annotation, we also provide an additional mapping to larger gene regulatory domains, centered on the transcriptional start site (TSS) of the surrounding genes (see Methods). Among the identified DMPs, the largest fraction corresponded to known sites affected by smoking^12^, which were replicated in our study (Table 1 and Fig. 3). These include, for example, probes mapping to the *AHRR-EXOC3* (10 DMPs), *ALPPL2-ALPI* (5 DMPs), *IER3-DDR1* (4 DMPs), *CNTNAP2* (3 DMPs), *CDKN1A* (2 DMPs), *F2RL3* (1 DMP) and *RPAP2-GLMN-GFI1* (1 DMP) loci. As previous studies were mainly restricted to active smokers, we verified that the changes in methylation are consistent with a genuine effect of smoking, when strictly selecting only the active smokers in the W5Y group (Supplementary Figure 5). Interestingly, the absolute average Δβ for the 58 DMPs was often larger in the S cohort compared to the B cohort (mean |Δβ| ± SEM, 0.061 ± 0.005 and 0.034 ± 0.004, respectively), possibly reflecting the different biological characteristics of the two cohorts, such as sex, HLA genotype or smoking load (estimated by the Pack-Year variable, PY). Indeed, for the W5Y class, the mean PY ± SEM was 14.66 ± 0.45 and 12.01 ± 0.30, for the S and B cohort, respectively.

**Table 1:** Summary of the 58 DMPs detected in blood of MS patients between W5Y *vs*. NS.

CpG ID	Chr.	Position	Direction	P-value	FDR	Δβ_S_	Δβ_B_	Gene	mapped TSS	distance to TSS	Known
cg04885881	chr1	11123118	−−	1.22E-07	0.00177	−0.0403	−0.0397		*SRM*	3036	Yes
cg25189904	chr1	68299493	−−	4.80E-06	0.0368	−0.0889	−0.065	*GNG12*	*GNG12*	342	Yes
cg09935388	chr1	92947588	−−	1.63E-07	0.00222	−0.174	−0.0497	*GFI1*	*RPAP2,GLMN,GFI1*	183066, 183054, 1922	Yes
cg06635952	chr2	70025869	++	3.31E-06	0.0279	0.0244	0.0206	*ANXA4*	*ANXA4,GMCL1*	56763,30903	Yes
cg26271591	chr2	178125956	−−	9.91E-09	0.000241	−0.0626	−0.0444	*NFE2L2*	*HNRNPA3,NFE2L2*	48665,3902	Yes
cg03329539	chr2	233283329	−−	6.40E-09	0.000164	−0.043	−0.0329		*ALPPL2,ALPI*	11776,37502	Yes
cg06644428	chr2	233284112	−−	1.12E-09	0.0000325	−0.0387	−0.0231		*ALPPL2,ALPI*	12559,36719	Yes
cg05951221	chr2	233284402	−−	4.53E-19	2.83E-14	−0.113	−0.0643		*ALPPL2,ALPI*	12849,36429	Yes
cg21566642	chr2	233284661	−−	1.75E-24	3.81E-19	−0.172	−0.102		*ALPPL2,ALPI*	13108,36170	Yes
cg01940273	chr2	233284934	−−	1.17E-23	1.70E-18	−0.12	−0.0631		*ALPPL2,ALPI*	13381,35897	Yes
cg21197336	chr3	193587490	−−	5.13E-06	0.0374	−0.0534	−0.0303		*OPA1,HES1*	276557,266442	Yes
cg04492193	chr4	8135668	++	5.65E-06	0.0399	0.0411	0.0211	*ABLIM2*	*AFAP1,ABLIM2*	194014,24767	No
cg03991871	chr5	368447	−−	1.21E-07	0.00177	−0.0668	−0.0364	*AHRR*	*AHRR,EXOC3-AS1,EXOC3*	64156,74810, 74824	Yes
cg23916896	chr5	368804	−−	3.16E-09	0.0000864	−0.0798	−0.0344	*AHRR*	*AHRR,EXOC3-AS1,EXOC3*	64513,74453, 74467	Yes
cg01899089	chr5	369969	−−	2.04E-07	0.00262	−0.0441	−0.0231	*AHRR*	*AHRR,EXOC3-AS1,EXOC3*	65678,73288, 73302	Yes
cg05575921	chr5	373378	−−	3.70E-27	1.62E-21	−0.22	−0.122	*AHRR*	*AHRR,EXOC3-AS1,EXOC3*	69087,69879, 69893	Yes
cg26703534	chr5	377358	−−	4.49E-13	2.45E-08	−0.0601	−0.0286	*AHRR*	*AHRR,EXOC3-AS1,EXOC3*	73067,65899, 65913	Yes
cg14817490	chr5	392920	−−	3.49E-10	0.0000117	−0.0616	−0.0392	*AHRR*	*AHRR,EXOC3-AS1,EXOC3*	88629,50337, 50351	Yes
cg17287155	chr5	393347	−−	4.45E-07	0.00512	−0.0097	−0.0173	*AHRR*	*AHRR,EXOC3-AS1,EXOC3*	89056,49910, 49924	Yes
cg25648203	chr5	395444	−−	2.64E-08	0.000502	−0.0523	−0.0276	*AHRR*	*AHRR,EXOC3-AS1,EXOC3*	91153,47813, 47827	Yes
cg21161138	chr5	399360	−−	3.46E-20	3.02E-15	−0.0742	−0.0508	*AHRR*	*AHRR,EXOC3-AS1,EXOC3*	95069,43897, 43911	Yes
cg24090911	chr5	400732	−−	2.79E-08	0.000508	−0.0424	−0.0327	*AHRR*	*AHRR,EXOC3-AS1,EXOC3*	96441,42525, 42539	Yes
cg14580211	chr5	150161299	−−	2.22E-07	0.00269	−0.0779	−0.0197	*SMIM3*	*SMIM3,IRGM*	3791,64784	Yes
cg06126421	chr6	30720080	−−	1.09E-19	7.96E-15	−0.144	−0.0726		*IER3,DDR1*	7748,131779	Yes
cg14753356	chr6	30720108	−−	9.43E-12	3.75E-07	−0.091	−0.0311		*IER3,DDR1*	7776,131751	Yes
cg24859433	chr6	30720203	−−	2.12E-07	0.00265	−0.0305	−0.0218		*IER3,DDR1*	7871,131656	Yes
cg15342087	chr6	30720209	−−	2.01E-10	0.00000731	−0.0335	−0.0223		*IER3,DDR1*	7877,131650	Yes
cg21091547	chr6	36645500	−−	1.89E-08	0.000375	−0.0517	−0.028	*CDKN1A*	*CDKN1A*	985	Yes
cg15474579	chr6	36645812	−−	1.27E-07	0.00179	−0.0572	−0.0196	*CDKN1A*	*CDKN1A*	673	Yes
cg10691866	chr7	65817282	−−	4.31E-06	0.0342	−0.0287	−0.0204	*TPST1*	*TPST1,KCTD7, RABGEF1*	147096,388359, 388436	Yes
cg21322436	chr7	145812842	−−	1.40E-12	6.80E-08	−0.0551	−0.0282	*CNTNAP2*	*CNTNAP2*	609	Yes
cg18804920	chr7	146572638	++	7.42E-06	0.0463	0.0325	0.0132	*CNTNAP2*			Yes
cg11207515	chr7	146904205	++	4.51E-06	0.0352	0.0633	0.0313	*CNTNAP2*			Yes
cg04172771	chr8	50892702	−−	1.40E-06	0.0142	−0.0334	−0.0171	*SNTG1*	*SNTG1*	68469	No
cg24838345	chr8	125737353	−−	2.61E-07	0.00308	−0.0264	−0.0205	*MTSS1*	*TATDN1,NDUFB9, MTSS1*	186027, 186009,3316	Yes
cg12075928	chr8	141801307	−−	1.17E-08	0.000269	−0.0352	−0.0522	*PTK2*	*AGO2,PTK2*	155588, 209949	Yes
cg26361535	chr8	144576604	−−	8.61E-08	0.00136	−0.029	−0.0236	*ZC3H3*	*MAFA,ZC3H3*	64027,47018	Yes
cg03450842	chr10	80834947	−−	6.27E-06	0.0428	−0.029	−0.0236	*ZMIZ1*	*ZMIZ1,PPIF*	6155,272285	Yes
cg16611234	chr11	58870075	−−	8.48E-07	0.00923	−0.0569	−0.0178		*FAM111B*	4633	Yes
cg19254163	chr11	60623782	−−	2.32E-06	0.0211	−0.0218	−0.019	*PTGDR2*	*PTGDR2*	337	Yes
cg11660018	chr11	86510915	−−	1.45E-06	0.0144	−0.0471	−0.0236	*PRSS23*	*PRSS23*	365	Yes
cg23771366	chr11	86510998	−−	6.41E-08	0.00108	−0.0408	−0.0242	*PRSS23*	*PRSS23*	282	Yes
cg14462402	chr11	120678447	−−	7.10E-06	0.045	−0.078	−0.0179	*GRIK4*	*GRIK4,TBCEL*	295979,216381	Yes
cg07986378	chr12	11898284	−−	4.00E-06	0.0324	−0.0819	−0.0377	*ETV6*	*ETV6,BCL2L14*	95496,325587	Yes
cg20059012	chr12	53613154	−−	1.39E-06	0.0142	−0.0398	−0.0316	*RARG*	*ITGB7,RARG*	12062,12881	Yes
cg27252467	chr13	19585665	++	2.70E-06	0.0236	0.052	0.0213	*LOC348021*	*TUBA3C*	170326	No
cg20303561	chr14	91881497	−−	2.58E-06	0.023	−0.0169	−0.0359	*CCDC88C*	*GPR68,CCDC88C*	161227,2623	Yes
cg05284742	chr14	93552128	−−	8.65E-07	0.00923	−0.0337	−0.0158	*ITPK1*	*CHGA,ITPK1*	162703,30019	Yes
cg23161492	chr15	90357202	−−	8.27E-10	0.0000258	−0.0677	−0.0283	*ANPEP*	*ANPEP*	891	Yes
cg03873392	chr16	10801987	++	6.81E-06	0.045	0.0414	0.0255		*TEKT5,NUBP1*	13184,35713	No
cg19572487	chr17	38476024	−−	1.68E-12	7.33E-08	−0.0507	−0.0519	*RARA*	*RARA,GJD3*	10578,44042	Yes
cg07251887	chr17	73641809	−−	5.61E-06	0.0399	−0.0489	−0.0171	*SMIM6; RECQL5*	*SMIM6*	706	Yes
cg03636183	chr19	17000585	−−	5.75E-23	6.28E-18	−0.11	−0.0686	*F2RL3*	*F2RL3*	914	Yes
cg25602603	chr19	22320744	++	3.48E-06	0.0287	0.0429	0.0261		*ZNF257,ZNF676*	85453,59008	No
cg03364381	chr21	43099460	++	7.08E-06	0.045	0.062	0.0356	*LINC00111*	*TMPRSS2,RIPK4*	219467,87805	No
cg01127300	chr22	38614796	−−	3.03E-06	0.0259	−0.0356	−0.0243		*MAFF,TMEM184B*	16907,54243	Yes
cg10293293	chrX	23761105	++	6.98E-06	0.045	0.0594	−0.008	*ACOT9*	*ACOT9*	284	No
cg24123432	chrX	105412256	−−	6.12E-06	0.0425	−0.0252	−0.0268		*SERPINA7*	130870	No

The CpG site is marked as known if present in reference^12^; Δβ: average methylation difference between W5Y and NS; TSS: transcription start site.

**Figure 4:**
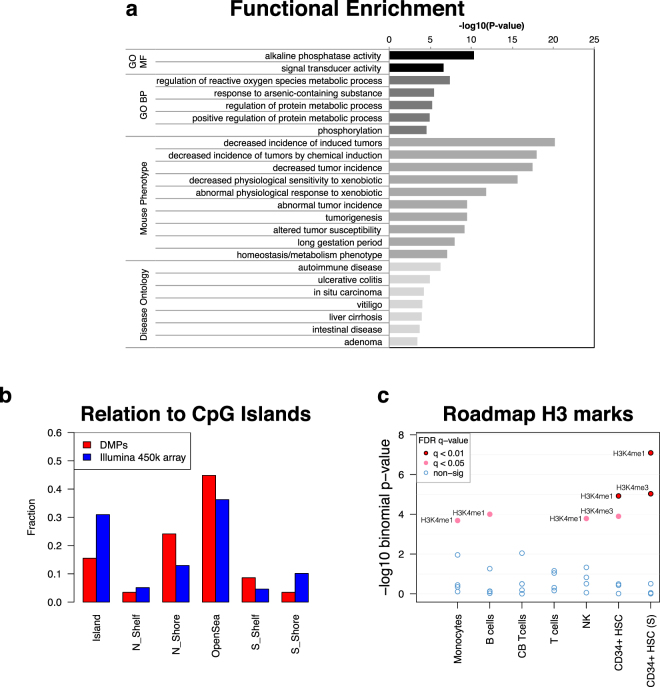
Functional annotation of DMPs. (**a**) GREAT analysis was performed to retrieve functional categories associated with DMPs. Up to top 10 categories per ontology group are shown (FDR < 0.05, fold-enrichment ≥2 and at least two gene hits), for the following groups: Gene Ontology Molecular Function (GO-MF), Gene Ontology Biological Process (GO-BP), Mouse phenotype and Disease Ontology. (**b**) For the annotation with respect to CpG islands, the relative fraction of positions located within each feature type is calculated for DMPs (red bars) and the entire 450k array (blue bar). (**c**) The enrichment −log_10_P-values for all H3 histone marks of the blood cell types from NIH Roadmap Epigenomics data was obtained with eFORGE, with default setting. Dots are colored according to the FDR and significant modifications are labeled. Abbreviations: CB, cord blood; HSC, Hematopoietic Stem Cells; S, single donor.

We next sought to investigate the physio-pathological relevance of the identified DMPs by performing functional annotation and ontology enrichment analysis. To this end, we defined an association rule between CpG positions and genes that considered gene regulatory domains (see Methods). Results are presented in Fig. 4. We observed relevant enrichment of terms related to metabolism of xenobiotics, including reactive oxygen species metabolism, de-phosphorylation and tumorigenesis (Fig. 4a). These terms likely reflect the detoxifying pathways activated by the chemicals contained in cigarette smoke, which are well known xenobiotics with carcinogenic activity. Interestingly, annotation with human disease ontology revealed enrichment for autoimmune diseases among the top categories, as several of the associated genes are linked to autoimmunity (Fig. 4a).

In addition to the above annotation, we performed further functional annotation of the smoking- associated (W5Y *vs*. NS) DMPs with respect to localization to CpG islands or regulatory regions reported from the ENCODE and NIH Roadmap Epigenomics projects, which include promoter marks, enhancer marks and DNase I Hypersensitivity sites (DHSs) from several cell types. The results revealed preferential localization outside CpG islands (Fig. 4b) and significant enrichment in regulatory regions of CD34+ hematopoietic stem cells, namely enhancers and promoters, identified by H3K4me1 or H3K4me3 histone marks respectively (Fig. 4c). Consistent with this observation, there was also a preferential enrichment in regions of accessible chromatin of CD34+ cells, as defined by DHSs (Supplementary Figure 6).

### Smoking induces DNA methylation and gene expression changes of the *AHRR* gene

**Figure 5:**
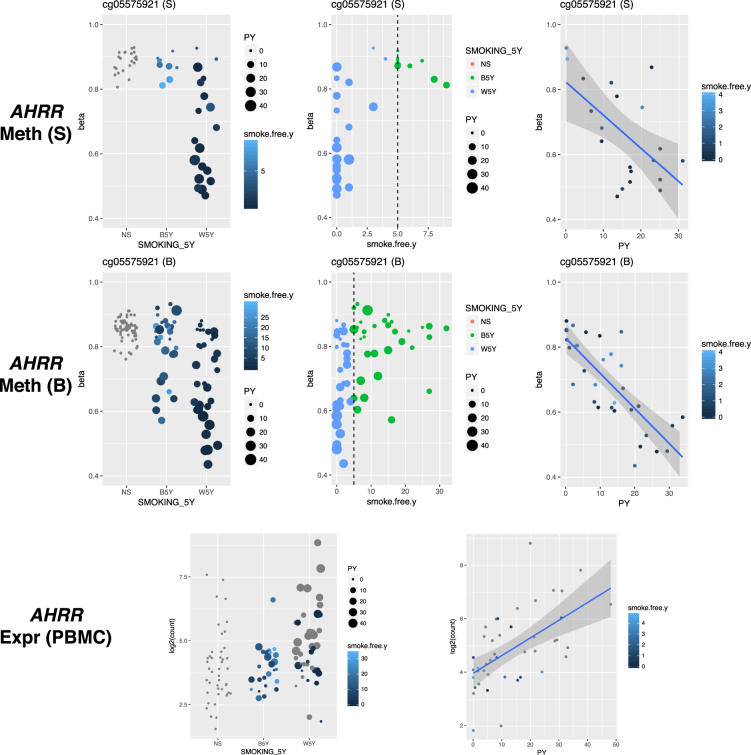
The effect of smoking is proportional to the smoking load and decrease as time since smoking cessation increase. DNA methylation is shown for the cg05575921 site, separately for the S and B cohorts. Methylation levels (β values) are shown in relation to the NS, B5Y and W5Y smoking categories (left panels), the time since smoking cessation (middle panels), and the Pack-Years (PY) in the W5Y group only (right panels). *AHRR* gene expression levels are shown for the PBMC cohort as normalized counts, in relation to the NS, B5Y and W5Y smoking categories and the Pack-Years in the W5Y group. Grey dots correspond to individuals in the W5Y group that were actively smoking at the date of sampling in the PBMC cohort.

We next aimed to examine the dynamics of smoking-induced alteration on DNA methylation levels and asked whether there is an effect of the time since smoking cessation on DNA methylation of the identified DMPs. Figure 5 shows the results for the top-associated marker of smoking cg05575921 (W5Y *vs*. NS P = 3.70 × 10^−27^), which is hypomethylated in the smoking group and lies within an intron of the *AHRR* gene. Notably, the hypomethylation is not exclusive for the individuals with certain risk factors (sex and HLA haplotype, data not shown), but the level of hypomethylation for the patients in the W5Y group is proportional to the smoking load, calculated as PY (Fig. 5, R^2^_S_ = 0.35, P_S_ = 0.006; R^2^_B_ = 0.50, P_B_ = 1.25 × 10^−11^). The change at this DMP is considerable (minimum observed β = 0.44 in the W5Y class compared to β = 0.76 in NS) but reversible after smoking cessation, and concordantly the average methylation value approaches the value observed in NS as smoke-free time after cessation increases. However, the time for restoration toward baseline DNA methylation appears to be influenced by the smoking load, as patients with a larger PY value display lower methylation values than patients with smaller PY but in similar smoke-free year range (Fig. 5). We observed similar findings for the majority of the other DMPs with genome-wide significant P-values (Supplementary Figures 7 and 8). We also asked if smoking induces transcriptional changes in the *AHRR* gene and indeed we verified that in PBMCs from patients with MS (PBMC cohort) the *AHRR* gene is upregulated in smokers as compared to non-smokers (P = 2.10 × 10^−3^ W5Y vs. NS, Fig. 5) and the average fold change increased when we considered as smokers only those individuals that reported smoking within the previous 12 months from the time of sampling (P = 3.76 × 10^−5^ CS vs. NS, Supplementary Figure 9). We also observed a positive exposure-response effect between PY and *AHRR* expression (Fig. 5, R^2^ = 0.33, P = 5.33 × 10^−5^). Among the genes listed in Table 1 and linked to the DMPs, only *AHRR* showed both significant differential expression and exposure-response relationship in PBMCs.

At the discovery phase with the cohort-specific and meta-analysis results, we used an association model on the M-values, therefore the calculated effect size is not directly interpretable. Thus, we fitted a beta regression model with variable dispersion in order to precisely model the effect of smoking for the MS patients in the larger B cohort at the cg05575921 site (see Methods and Table 2). As expected, the PY effect was highly significant (P < 2 × 10^−16^), and the smoking group W5Y remained significant after correcting for PY (P = 0.008). When we predicted the methylation levels for MS patients in the W5Y category with PY = 15, we obtained a β = 0.67, corresponding to a Δβ = −18% as compared to NS. In conclusion, smoking affects DNA methylation in patients with MS at specific genome-wide significant CpGs in an exposure-dependent manner. The effect - mostly hypomethylation - is more pronounced in MS patients that are smokers or ceased smoking less than 5 years prior to the time of sampling, while methylation levels are restored to the levels of non-smokers in MS patients who quit smoking five years or more prior to the time of sampling, but with dynamics influenced by the smoking load. Specifically, we observed demethylation of the AHRR gene and increased expression after smoking.

**Table 2:** Summary of the beta regression.

MS-only model *(μ* ~ PY + Smoking, *ϕ* ~ Smoking)			
**MS/HC model** ***(μ*** **~ PY** + **MS** + **Smoking** + **PY:MS**, ***ϕ*** **~ Smoking)**			
*Regression parameters for the mean (μ)*			
**Predictor**	Effect*	**95% CI**	**P-value**
Pack-Year (PY)	−0.050	−0.061–−0.038	<2 × 10^−16^
Smoking B5Y	−0.014	−0.197–0.170	0.88
Smoking W5Y	−0.295	−0.512–−0.078	7.70 × 10^−3^
***Regression parameters for the precision (ϕ)***			
**Predictor**	**Effect**	**95% CI**	**P-value**
Smoking B5Y	−1.380	−1.969–−0.792	4.33 × 10^−6^
Smoking W5Y	−1.422	−2.009–−0.835	2.04 × 10^−6^
***Regression parameters for the mean (μ)***			
**Predictor**	Effect*	**95% CI**	**P-value**
Pack-Year (PY)	−0.023	−0.031–−0.015	1.17 × 10^−8^
MS	0.047	−0.026–0.119	0.21
Smoking B5Y	0.065	−0.048–0.179	0.26
Smoking W5Y	−0.326	−0.462–−0.191	2.24 × 10^−6^
PY:MS interaction	−0.027	−0.037–−0.016	3.86 × 10^−7^
***Regression parameters for the precision (ϕ)***			
**Predictor**	**Effect**	**95% CI**	**P-value**
Smoking B5Y	−1.086	−1.504–−0.668	3.59 × 10^−7^
Smoking W5Y	−1.556	−1.963–−1.148	7.30 × 10^−14^

*Effect: indicates the change in log-odds for the DNA methylation estimate (logit link).

### Is there any differential effect of smoking between MS patients and healthy controls?

Next, we asked whether the disease-related processes could alter the effect of smoking. Since the DMPs could lie within regulatory elements of genes that are located at a considerable distance, we considered mapping to regulatory domains (see Methods) to explore the relevance of the reported DMPs with MS. Thus, we verified that several of the DMPs listed in Table 1 map to the regulatory domains of genetic risk loci for MS or genes involved in MS or its animal model, *i.e*. Experimental Autoimmune Encephalomyelitis (EAE). Examples of a direct involvement either in autoimmunity, or MS susceptibility and pathogenesis, include *GFI1*^20,21^, *ZMIZ1*^2,22^ and *NFE2L2*^23^. Moreover, *CNTNAP2*^24^ and *DDR1*^25^ are involved in demyelination/remyelination processes and could be indirectly linked to MS, while *AHRR*^26^, *ITGB7*^27^, *PPIF*^28^ and *GPR68*^29^ have been studied in the context of EAE. Ultimately, 12 out of the 58 reported DMPs lie within an LD block containing at least one SNP considered as an MS risk according to the GWAS catalog. Although this enrichment is not significant as compared to a random expectation (data not shown), we hypothesized that the chronic inflammatory nature of the MS disease could potentially influence the DNA methylation changes observed in blood.

**Figure 6:**
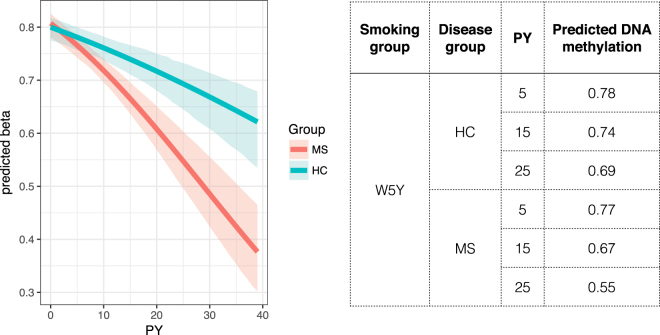
The effect of the pack-years interacts with the disease. The fitted β values were obtained as a function of Pack-Years (PY) for MS (red) and controls (blue) in the W5Y smoking category, by using a beta regression model, as indicated in the main text. 95% confidence levels were estimated by bootstrapping with 1000 replications and are shown as shaded areas.

Thus, we analyzed genome-wide DNA methylation in a matched cohort of healthy controls stratified for the corresponding NS, B5Y and W5Y categories. Since the strict selection criteria of the S cohort did not allow inclusion of healthy control group with adequate size, the results presented in this section were obtained exclusively from the B cohort. In order to address the general cohort integrity, we first assessed the enrichment for known smoking associated CpGs (Supplementary Figure 10), confirming that the effect on DNA methylation is also detectable in the healthy control group, although with limitation intrinsically given by the sample size. We modeled the methylation level of the aforementioned top marker cg05575921 by including smoking load (PY), disease group (HC/MS) and smoking category as predictive variables, using beta regression with variable dispersion (see Methods). We found that the smoking group W5Y remained significant (P = 2.24 × 10^−6^), and that the smoking load was highly significant (P = 1.17 × 10^−8^) as well as its interaction with the disease group (P = 3.86 × 10^−7^). Age, sex and therapy in the 3 months prior to sampling were not significant and therefore were excluded from the final model. Similar conclusions might be drawn for other DMPs of the *AHRR* gene (Supplementary Table 2). We present evidence that, while the disease status does not affect DNA methylation, the presence of the disease might exacerbate the effect of the smoking load, by enhancing the extent of hypomethylation (Table 2 and Fig. 6). The variability is also affected, as we find that the dispersion is significantly increased in the smoking categories as compared with NS (Table 2 and Fig. 5). For example, when the methylation levels were predicted using this model, for an individual in the W5Y group smoking 15 PY, the predicted DNA methylation at the cg05575921 locus was 0.67 for MS cases, and 0.74 for controls. Figure 6 shows the fitted effect of PY for cases and controls, and graphically shows how the smoking load statistically interacts with the disease.

## Discussion

In this study we selected two separate cohorts from the EIMS project, whose main goal is to contribute to an increased understanding of the factors causing MS, with a focus on environmental/life-style and genetic factors and their interaction. We performed cohort-specific and meta-analysis studies, in order to assess the effect of smoking, in the context of a chronic inflammatory disease. The rationale behind this study is based on the consistent body of evidence involving smoking in MS pathogenesis. Firstly, epidemiological studies have established a strong association between smoking and increased susceptibility risk, disease progression and clinical disability^6^. A gene-smoking interaction has also been observed, as the effect of the major determinant of genetic risk, HLA genotype, has been shown to be modified by smoking^10^. Secondly, from a mechanistic point of view, smoking is known to induce lung tissue inflammation and promote pro-inflammatory pathways^30^, processes that have been implicated in the development of neuroinflammatory responses in the central nervous system^6,31^. In this context, epigenetic processes (DNA methylation) are likely to contribute to the response to environmental exposures (smoking), by mediating or altering the impact of the external trigger on the gene expression networks and cellular function during neuroinflammation. Finally, it has been extensively demonstrated that blood DNA methylation is affected by cigarette smoke and that an exposure-response relationships of smoking load and time since cessation could further explain DNA methylation levels^11,12^.

Motivated by the above considerations, we aimed at analyzing the impact of smoking on DNA methylation in blood from MS patients. With respect to the smoking phenotype, we stratified the samples with an epidemiologically-educated criterion, stating that an increased risk for MS exists for individuals that ceased smoking in a period less than five years^7^. Moreover, a large-scale meta-analysis of the effect of smoking^12^ recently confirmed that the DNA methylation levels of many of the smoking-related DMPs returned to the basal levels within five years of smoking cessation. Accordingly, in our study we observed an exposure-response effect and a restoration to the levels of non-smokers for the top associated DMPs. Despite the five-year window as a reasonable cut-off in our study, we found individual sources of variability and importantly we observed that the time for the reversal of the methylation levels could be influenced by the extent of smoking. Therefore, a more precise and accurate individualized assessment should be performed on larger cohorts of patients or in a longitudinal scenario to address this issue.

A limitation of this study is the relatively small sample size, which likely increases the number of false negatives and results in narrow generalization. On the other hand, we controlled the number of false positives by using both an FDR and an effect size condition, resulting in identification of DMPs, which genuinely overlapped with previous reports^11,12^. While it is tempting to speculate that the novel sites (see Table 1) could be specific for the MS context, caution should be taken when interpreting the results, as other unknown sources of variability could influence the reported differences. Nevertheless, our study highlights that a differential effect of the smoking load on DNA methylation is observed in patients as compared to healthy controls, an observation that deserves further studies, as we lack a complete understanding of the molecular events that mediate the risk of developing MS conferred by cigarette smoking. Although it is demonstrated that environmental and lifestyle factors interact with immune response genes^6^, it has not been fully revealed how the autoimmune reaction is fine-tuned by the presence of those risk factors in a mechanistic way. Therefore, further support is needed for the generalization of the findings, especially to rule out, in larger cohorts, that the effect is not inherently biased by the fact that smoking might be more common among MS patients than healthy controls (higher PY).

Another aspect that would require further investigation is the fact that we generally observed larger effect sizes (ΔM value) in the smaller cohort (S), albeit with greater standard error, corresponding to larger average difference in methylation (Δβ), as compared to the larger cohort (B). If we exclude merely technical and systematic reasons (quality of the arrays, different facilities, systematic biases in the phenotyping etc.), it is worth speculating whether this observation can be linked to the “risk” characteristics of the samples in the S cohort, which included only women carrying the MS- and smoking-interacting HLA-DRB1*15:01 risk genotype and lacking the protective *HLA-A2* genotype. Although the major cell types did not appear to be markedly affected, it cannot be excluded that the different biological characteristics of the two cohorts, including for example inflammation, are linked to different proportions of certain subsets of cells, which in turn would influence the detected methylation signal. As an example in support of this possibility, we observed that a region in the gene body of *GFI1* containing a significant DMP is more demethylated in smokers of the S cohort, compared to the B cohort. *GFI1* is a transcriptional repressor that regulates the differentiation of several hematopoietic cell types^32^ and its downregulation is crucial for T helper (Th) 17 differentiation^21^. Differences in expression/methylation of this gene are therefore likely among subpopulations of blood cells and they could be responsible for the observed heterogeneity. Alternatively, a simpler explanation would be that the higher smoking load in the S cohort contributes to a stronger average Δβ difference, or that the exposure to passive smoking of some non-smokers in the B cohort constitutes a confounding factor. All the above considerations highlight the importance of controlling for relevant covariates in the experimental design and at the analysis phases, to account for possible sources of additional variation. Indeed, the average effect size in differential methylation is modest, with only a few of the 58 CpGs showing average difference >10%, although the spread of the individual methylation levels in smokers is high. This might conceivably reflect both the exposure-response relationship and other biological sources of variation.

The AhR pathway is involved in the metabolism of xenobiotics and it is activated by environmental pollutants, including dioxins contained in cigarette smoke^33^. Furthermore, the AhR pathway exerts environmental control on the immune response, in particular in neuroinflammation^34,35^, and it is known as a regulator of the Th17 and regulatory T cell subsets^36,37^. Further relevance as a therapeutic target for immunological diseases is shown by the recent finding that Laquinimod, a drug being evaluated for the treatment of MS, activates the AhR pathway and upregulates CYP1A1 and AHRR^26^. The *AHRR* gene, one of the top demethylated loci after smoking in our investigation as well as previous studies, is activated by the AhR/ARNT heterodimer and its activity inhibits AhR function in a negative feedback regulation that involves a competition with AhR for heterodimerizing with ARNT and binding with the responsive DNA sequences^38^. The antagonism of the AhR protein leads simultaneously to an increased expansion of CD34+ cells and decreased AHRR levels^39^. Interestingly, we observed enrichment of smoking-related DMPs on enhancers of CD34+ together with decreased methylation and increased expression of *AHRR*. Although we did not evaluate the connection between *AHRR* methylation and expression in matched RNA and DNA samples obtained concurrently from the same individuals, we speculate that there might be a link between the deregulated AHRR/AhR activity, and the differential number of CD34+ cells after smoking. Indeed, it has previously been observed that smoking is associated with decreased circulating CD34+ cells^40^. Furthermore, it remains an open question whether the inflammation in MS is influencing the mobilization of CD34+ cells to the peripheral blood, as some studies show that Natalizumab increases the proportion of blood CD34+ cells in MS patients and that response to the treatment is associated with their mobilization^41^.

In conclusion, smoking might act as an environmental epigenetic modifier that is able to hamper the cellular regulatory networks, which are in essence built by interactions between genes and are remodeled in association with the onset of MS and its natural history. Both from a public health and pathogenetic perspective, the impact of smoking has a fundamental importance in the understanding and the management of MS, therefore the findings presented here provide clues for further study of the connection between the environment and the reversible epigenetic changes observed in MS patients that associate with smoking.

## Conclusions

We verified that smoking has a genome-wide significant effect on blood DNA methylation in MS patients, and that the effect is especially evident in current smokers and patients that stopped smoking in a period of less than 5 years prior to sampling. Although the effect does not seem to be confounded by the major cell types’ proportions, we found an interesting association with CD34+ regulatory sites. We analyzed in detail the *AHRR* gene, whose expression is increased in smokers and shows hypomethylation at several CpG sites. For the top significant site (cg05575921), we thoroughly modeled the effect of the smoking load and we suggested an interaction with the disease, conceivably indicating a connection between a modifiable risk factor, an epigenetic modification and the pathogenetic mechanisms.

## Methods

### Ethics, consent and sample collection

The Regional Ethical Review Board in Stockholm approved this study (# 04-252/1-4, 02-548 and 2009/2107-31/2) and methods were carried out in accordance with institutional guidelines on human subject experiments. Informed consent was obtained from all subjects. All samples were collected between 2005 and 2009 and are part of the large and unique Epidemiological Investigation of Multiple Sclerosis (EIMS) cohort in Sweden, comprising MS cases and controls matched by age, sex, and residential area^7^. Self-reported smoking information (cigarettes, cigars and pipes) was acquired from the EIMS questionnaire. DNA was extracted from whole blood.

### Study cohorts

Description of the cohorts is shown in Supplementary Table 1. The Selected cohort (S) consisted of 50 patients, stratified by considering the time they reported the last smoking event, prior to the time of sampling, *i.e*. within 5 years (W5Y, n = 19), 5 years and beyond (B5Y, n = 9), and never-smokers (NS, n = 22). Importantly, all individuals included in this cohort are Swedish females in the age of 26–59 years, carrying the genetic risk for MS, i.e. carrier of HLA-DRB1*15:01 risk variant and non-carrier of *HLA-A2* protective variant. All NS reported no exposure to passive smoking and one smoker reported the concomitant use of cigars. Disease duration spanned from 0–10, 0–7 and 0–5 years prior to sampling for W5Y, B5Y and NS, respectively.

The Broad cohort (B) consisted of 132 MS patients and 135 healthy controls (HC) divided into W5Y (33 cases and 34 controls), B5Y (34 cases and 31 controls) and NS (65 cases and 70 controls). Male and female Swedish individuals in the age of 16–66 years were included and they were not selected on the basis of their HLA genotype. 62 NS (30 cases and 32 controls) reported past exposure to passive smoke and 8 smokers (3 cases and 5 controls) reported the use of cigars or pipes. For MS cases, disease duration spanned from 0–30, 0–31 and 0–28 years prior to sampling for W5Y, B5Y and NS, respectively.

The peripheral blood mononuclear cell (PBMC) cohort consisted of 113 patients with MS or Clinically Isolated Syndrome included in a biobank of samples collected between 2001 and 2010, at the Neurology Clinic of the Karolinska University Hospital, Solna, Sweden. Patients were categorized into W5Y (n = 43), B5Y (n = 26) and NS (n = 44). Male and female individuals were included and they were not selected on the basis of their HLA genotype.

For all cohorts, the amount of smoking was quantified with the pack-year (PY) variable, calculated as $PY=(cigarettes\,smoked\,per/day20)\ast years\,as\,smoker$, assuming 20 cigarettes per pack.

### Methylation analysis

*S cohort*. 500 ng of bisulfite converted DNA (EZ DNA Methylation kit, Zymo research) was amplified, fragmented and hybridized to Illumina Infinium Human Methylation450k Beadchip using standard protocol at BEA core facility (Karolinska Institutet). The samples were randomly assigned to eight BeadChips with technical replicates and processed in one run. Three samples were technically replicated in pairs. BeadChips were scanned using an iScan and raw IDAT files were generated and processed in R with the minfi package^42^. We performed quality control with the shinyMethyl package^43^. We first excluded one failing sample on the basis of the control probe profiles. We then normalized the intensities and calculated methylation estimates (β values) using the Functional Normalization algorithm^44^. We excluded: probes with a minfi detection P-value > 0.01 in at least 10% of the samples; probes with common single nucleotide polymorphisms (SNPs) at the single base extension or at the CpG interrogation sites, as reported in the IlluminaHumanMethylation450kanno.ilmn12.hg19 annotation package (dbSNP 137); and cross-reactive probes as reported previously^45^. Phenotypic variables, cell type fraction estimates (see below) and technical variables (Beadchip and Array) were checked for association with the scores from a Principal Component Analysis, in order to identify covariates to include in our association model. We restricted our analysis to 436,999 probes (out of 485,512).

*B cohort*. 500 ng of bisulfite converted DNA (EZ DNA Methylation kit, Zymo research) was amplified, fragmented and hybridized to Illumina Infinium Human Methylation450k Beadchip using standard protocol at Johns Hopkins University School of Medicine. The samples were randomly assigned to three plates and 24 BeadChips and processed and analyzed as above. For PCA analysis, phenotypic variables, cell type fractions estimates (see below) and technical variables (plate, Beadchip and Array) were checked for association with the scores from a Principal Component Analysis, in order to identify covariates to include in our association model. We restricted our analysis to 437,034 probes (out of 485,512).

All bioinformatics analyses were performed in R, unless otherwise indicated.

### Cell type composition estimation

We estimated the blood cell type proportions (CD4+ T cells, CD8+ T cell, B cells, NK cells, monocytes and granulocytes) using the reference-based Houseman method^18^ in the minfi package for each cohort separately. The method makes use of methylation profiles obtained from isolated blood populations^46^ to deconvolute the heterogeneous signal from blood.

### Differential Methylation

Differentially methylated positions (DMPs) were obtained using multiple linear regression with limma^47^ on M-values, obtained by logit transformation of the β values. Infinite M-values were substituted with the minimum/maximum value of the finite M-value for the corresponding sample. We modeled the association between DNA methylation and smoking status (NS, W5Y, B5Y), in the presence of covariates. Probes were annotated with the IlluminaHumanMethylation450kanno.ilmn12.hg19 package and a β value change was calculated as the difference between the average group values.

For the S cohort, we included as covariates the age (years) and three Surrogate Variables (SVs) estimated with the sva method^48^, in order to control for unwanted variation. As an alternative to the inclusion of the SVs, another model was fitted including the age and the estimated cell type proportions (CD4+ T cells, CD8+ T cell, B cells, NK cells and monocytes). To account for the presence of technical replicates, we estimated the consensus correlation as a robust average of the individual correlation values obtained by fitting a mixed linear model individually for each gene, and then using this value in the linear model step as within-block correlation. Coefficients, P-values and FDRs (Benjamini-Hochberg) were obtained for the contrasts W5Y *vs*. NS, W5Y *vs*. B5Y and B5Y *vs*. NS. For the B cohort, we performed separate analyses for the healthy controls and the MS cases. We included as covariates the age (years), the sex, the passive smoking (no/yes) and three SVs, in order to control for unwanted variation. As an alternative to the inclusion of the SVs, another model was fitted including the age, the sex, the passive smoking and the estimated cell type proportions (CD4+ T cells, CD8+ T cell, B cells, NK cells and monocytes). Coefficients, P-values and FDRs (Benjamini-Hochberg) were obtained for the contrasts W5Y *vs*. NS, W5Y *vs*. B5Y and B5Y *vs*. NS.

### Meta-analysis

We performed meta-analysis with METAL^49^, using inverse-variance weighting. Briefly, individual coefficients from the limma analysis (i.e. the effect size estimates on the M-value scale) were combined using their estimated standard errors as weights in order to obtain and an overall P value. The FDR was obtained with the Benjamini-Hochberg procedure.

### GSEA

Gene Set Enrichment Analysis (GSEA) was performed using the package fgsea^50^ and the set of 62 CpG sites from Gao *et al*. as a gene set^11^. These sites were associated with smoking at least by three studies. We ranked all the analyzed sites for the three contrasts (W5Y *vs*. NS, W5Y *vs*. B5Y and B5Y *vs*. NS.) using the respective −log_10_P values, and the significance was assessed with 10,000 permutations.

### Gene regulatory domains and functional annotation

In order to map probes to genes in a broader way and also obtain a gene-based summary measure, we assigned the probes to large gene regulatory domains, similarly to the GREAT approach^51^. We obtained the subset of UCSC known genes, and to obtain transcription start sites we considered the canonical isoform as taken from the knownCanonical table of the UCSC knownGene track. Then, we constructed gene regulatory domains with the “basal plus extension rule” (constitutive 5.0 kb upstream and 1.0 kb downstream, up to 500 kb max extension. GREAT^51^ was used to discover functional categories associated with DMPs. The enrichment for known cell type-specific DNase 1 hypersensitive sites and histone modifications was performed with eFORGE^52^.

### Beta regression

We performed beta regression as implemented in the package betareg^53^. This model is especially suitable for a response variable that assumes values in the unit interval (0, 1) and in presence of heteroskedasticity. The methylation estimates from the arrays were modeled with a variable dispersion beta regression. For the regression with the MS-only cases, we included the smoking group (NS, B5Y and W5Y) and the PY regression parameters for the mean (*μ*) and the smoking group as regression parameters for the precision (*ϕ*). When considering all MS and HC subjects, we included the smoking group (NS, B5Y and W5Y), the PY, the case/control variable and its interaction with PY as regression parameters for *μ*, and the smoking group as regression parameters for the *ϕ*. Default link functions were used (logit for *μ* and log for *ϕ*).

### RNA-Seq analysis

A matrix with gene expression counts in PBMCs was obtained from James *et al*. (submitted). Counts were processed and normalized with DESeq2^54^ and a P-value for differential expression was obtained for smokers (W5Y or current smokers) vs. non-smokers.

### Bisulfite pyrosequencing

Bisulfite pyrosequencing was performed for technical validation. Primers were designed using PyroMark Design software (Qiagen), and sequences are presented in Supplementary Table 3. Three DMPs; cg05575921 (*AHRR*), cg21566642 (*ALPPL2*) and cg06126421 (*IER3/DDR1*), including additional adjacent CpGs (see Supplementary Figure 1), were selected for validation from the S cohort. Genomic DNA (500 ng) was treated with sodium bisulfite (Methylation gold Bisulfite Kit, Zymo) and subsequently 1 µl of converted DNA (~10 ng) was applied as template in the PCRs performed with the PyroMark PCR Kit (Qiagen). The entire PCR product and 4 pmol of the respective sequencing primer, and streptavidin sepharose high performance beads (GE Healthcare), were used for pyro-sequencing performed with the PSQ 96 system and the PyroMark Gold Q96 Reagent Kit (Qiagen). The PyroMark CpG software 1.0.11 served for data analysis.

### Availability of data

DNA methylation data are available on GEO under the accession numbers GSE60655 for the S cohort. The data from the B and PBMC cohorts are not publicly available at the submission date due to non-disclosure agreements for independent manuscripts, but are available from the corresponding author on reasonable request.

## Electronic supplementary material

Supplementary figures

Supplementary table 1

Supplementaty table 2

Supplementary table 3
