# Author Correction: Smoking induces DNA methylation changes in Multiple Sclerosis patients with exposure-response relationship

**DOI:** 10.1038/s41598-018-22686-y

**Published:** 2018-03-07

**Authors:** Francesco Marabita, Malin Almgren, Louise K. Sjöholm, Lara Kular, Yun Liu, Tojo James, Nimrod B. Kiss, Andrew P. Feinberg, Tomas Olsson, Ingrid Kockum, Lars Alfredsson, Tomas J. Ekström, Maja Jagodic

**Affiliations:** 1 Department of Clinical Neuroscience, Center for Molecular Medicine, Karolinska Institutet, Karolinska University Hospital, Stockholm, Sweden; 2 Department of Biochemistry and Molecular Biology, The Ministry of Education Key Laboratory of Metabolism and Molecular Medicine, School of Basic Medical Sciences, Fudan University, Shanghai, China; 3 State Key Laboratory of Medical Neurobiology, Fudan University, Shanghai, China; 4 Center for Epigenetics and Departments of Medicine, Biomedical Engineering, and Mental Health, Johns Hopkins University, Baltimore, MD USA; 5 Institute of Environmental Medicine, Karolinska Institutet, Stockholm, Sweden

Correction to: *Scientific Reports* 10.1038/s41598-017-14788-w, published online 06 November 2017

This Article contains errors in the Methods section, under subsection ‘Availability of data’.

“DNA methylation data are available on GEO under the accession numbers GSE60655 for the S cohort. The data from the B and PBMC cohorts are not publicly available at the submission date due to non-disclosure agreements for independent manuscripts, but are available from the corresponding author on reasonable request.”

should read:

“DNA methylation data are available on GEO under the accession numbers GSE43976 for the S cohort. The data from the B and PBMC cohorts are not publicly available at the submission date due to non-disclosure agreements for independent manuscripts, but are available from the corresponding author on reasonable request.”

