# Supplementary material for: Smoking induces DNA methylation changes in Multiple Sclerosis patients with exposure-response relationship
Source: Sci Rep. 2017 Nov 6;7:14589. doi: 10.1038/s41598-017-14788-w (PMC5674007; doi:10.1038/s41598-017-14788-w)
Supplement: Supplementary file 1 — Supplementary figures [file 41598_2017_14788_MOESM1_ESM.pdf]

Supplementary Figures

for manuscript

“Smoking induces DNA methylation changes in Multiple Sclerosis patients with exposure-response relationship”

Francesco Marabita, Malin Almgren, Louise K Sjöholm, Lara Kular, Yun Liu, Tojo James, Nimrod B Kiss, Andrew P Feinberg, Tomas Olsson, Ingrid Kockum, Lars Alfredsson, Tomas J Ekström, Maja Jagodic

Supplementary figure 1. Technical validation with bisulfite pyrosequencing. Three CpGs were validated with bisulfite pyrosequencing on DNA samples of the patients in the S cohort. All the CpGs in the PCR amplicon are shown and an asterisk indicates the CpG corresponding to the array probe, i.e. cg05575921 (AHRR), cg21566642 (ALPPL2) and cg06126421 (IER3). All differences in DNA methylation fractions between W5Y and NS are significant ($p<0.001$, unequal variances T test).

Supplementary figure 2. Estimation of cell type proportions with a reference-based method for the S and B cohort. We estimated the cell type composition with the Houseman reference-based algorithm. The proportions of granulocytes, monocytes, B cells, natural killer cells, CD4 T cells and CD8 T cells are shown as boxplots (A-B). The scatterplots (C-D) show the P-values for the indicated contrasts with the inclusion of the estimated proportions in the association model (y axis) or as presented in the main text with the adjustment for the SVs (x axis). The points are colored using the local density and the Pearson correlation is shown on the bottom right of the plot.

Supplementary figure 3. Comparison with HLA-adjusted model for the B cohort. The scatterplots show the P-values for the indicated contrasts. Values on the x axis correspond to the values from the Surrogarte Variable-adjusted analyses as presented in the main text, while values on the y result from the explicit adjustment for the HLA (“risk” vs. “no risk”). “Risk” patients are carriers of the *HLA-DRB1*15:01* allele (DR15+/+ or DR15+/-) and the non-carriers of the *HLA-A*02* allele (A2-/-). The points are colored using the local density and the Pearson correlation is shown on the bottom right of the plot.

Supplementary figure 4. “Concordance at the top” plots. For the *i* top-ranked CpGs of each list for the S and B cohort, concordance is defined as the fraction of the *i* probes in common between the S and B lists. This concordance is plotted as a function of *i*, for the ranked CpG lists obtained from the contrasts W5Y *vs.* NS (red), W5Y *vs.* B5Y (blue) and B5Y *vs.* NS (black). The inset shows an enlargement for the first 200 probes.

Supplementary figure 5. The effect of smoking on DNA methylation for active smokers. DNA methylation is shown for seven selected and replicated loci affected by smoking, separately for the S and B cohorts. One CpG per gene locus is shown, Methylation levels (β values) are shown in relation to the NS and W5Y smoking categories. The W5Y category was further stratified in active and non-active smokers, considering individual that reported smoking <1 year from the time of sampling as active smokers.

Supplementary figure 6. Functional annotation of DMPs, with respect to DHSs. The enrichment –$\log_{10}$P-values for the DHSs of the blood cell types from Encode (A) or NIH Roadmap Epigenomics (B) data was obtained with eFORGE, with default setting. Dots are colored according to the FDR and significant enrichments are labeled.

Supplementary figure 7. The 58 DMPs for the S cohort. The methylation levels (β values) for the 58 DMPs are shown in relation to the NS, B5Y and W5Y smoking categories or the time since smoking cessation.

Supplementary figure 8. The 58 DMPs for the B cohort. The methylation levels (β values) for the 58 DMPs are shown in relation to the NS, B5Y and W5Y smoking categories or the time since smoking cessation.

Supplementary figure 9. Gene expression of the AHRR gene in the PBMC cohort. *AHRR* gene expression levels are shown for the PBMC cohort as normalized counts. On the panels on the left, smoking is stratified as NS, B5Y and W5Y, while on the right smoking is stratified as follows: NS, never smokers; PS, patients that reported smoking >1 year from the time of sampling; CS, patients that reported smoking <1 year from the time of sampling. Grey dots mark individuals who reported smoking at the time of sampling. The PY-expression scatterplot is shown only for the patient in the W5Y or CS categories, respectively.

Supplementary figure 10. Enrichment of known smoking-affected CpGs for Healthy Controls samples. A GSEA approach was used to check the enrichment for known smoking-affected CpG. The green line on the top panels shows the Enrichment Score (ES). Vertical ticks mark the location of the 62 CpG from Gao *et al*. within the list of the probes ranked by decreasing significance in healthy controls and the bottom panels show their corresponding normalized $-\log_{10}P$ values, for the comparisons W5Y *vs*. NS, W5Y *vs.* B5Y and B5Y *vs.* NS, respectively.

Supp. Fig. 1

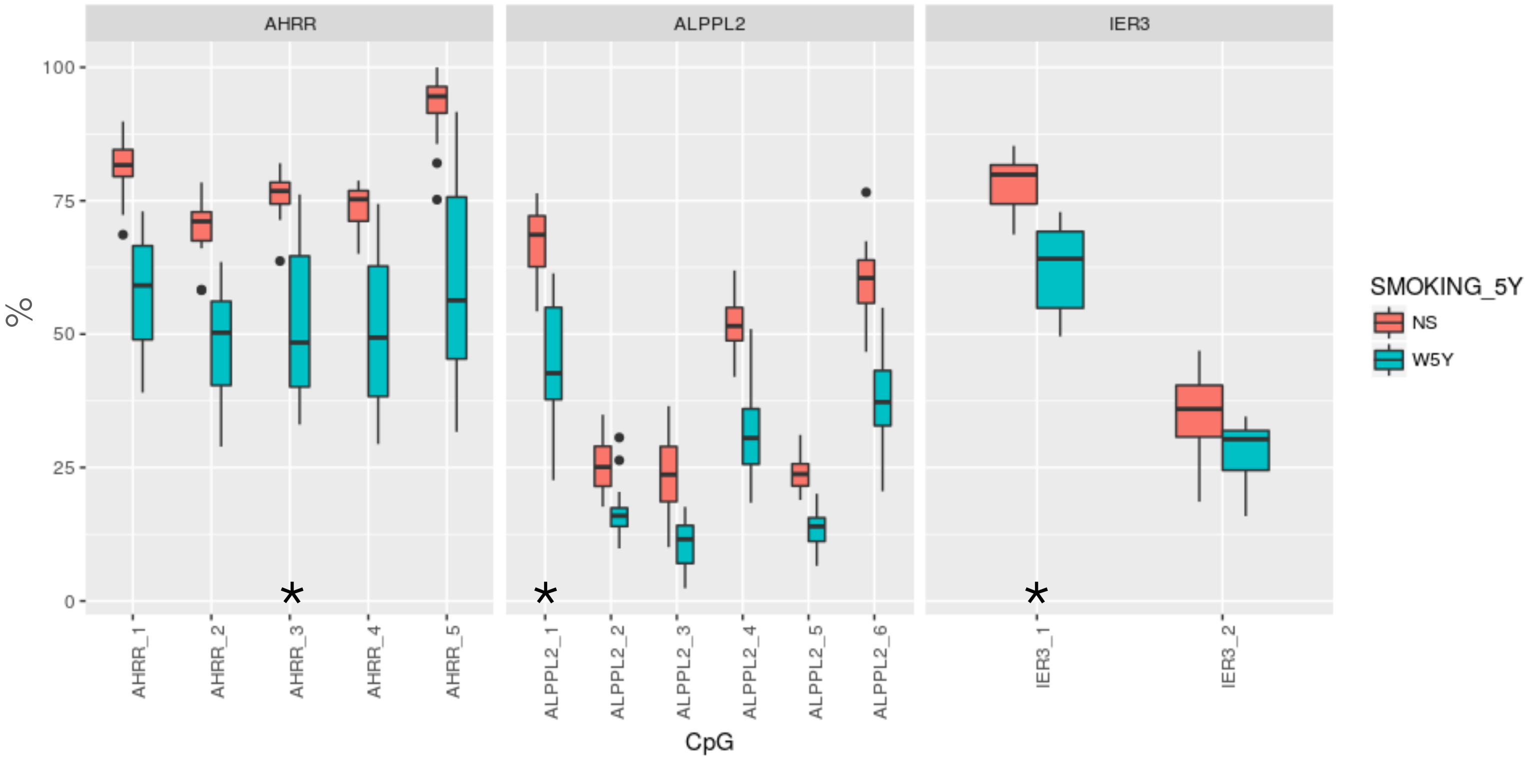

AHRR
ALPPL2
IER3
100
75
50
25
0
%
AHRR_1
AHRR_2
AHRR_3
AHRR_4
AHRR_5
ALPPL2_1
ALPPL2_2
ALPPL2_3
ALPPL2_4
ALPPL2_5
ALPPL2_6
IER3_1
IER3_2
CpG
SMOKING_5Y
NS
W5Y

Supp. Fig. 2

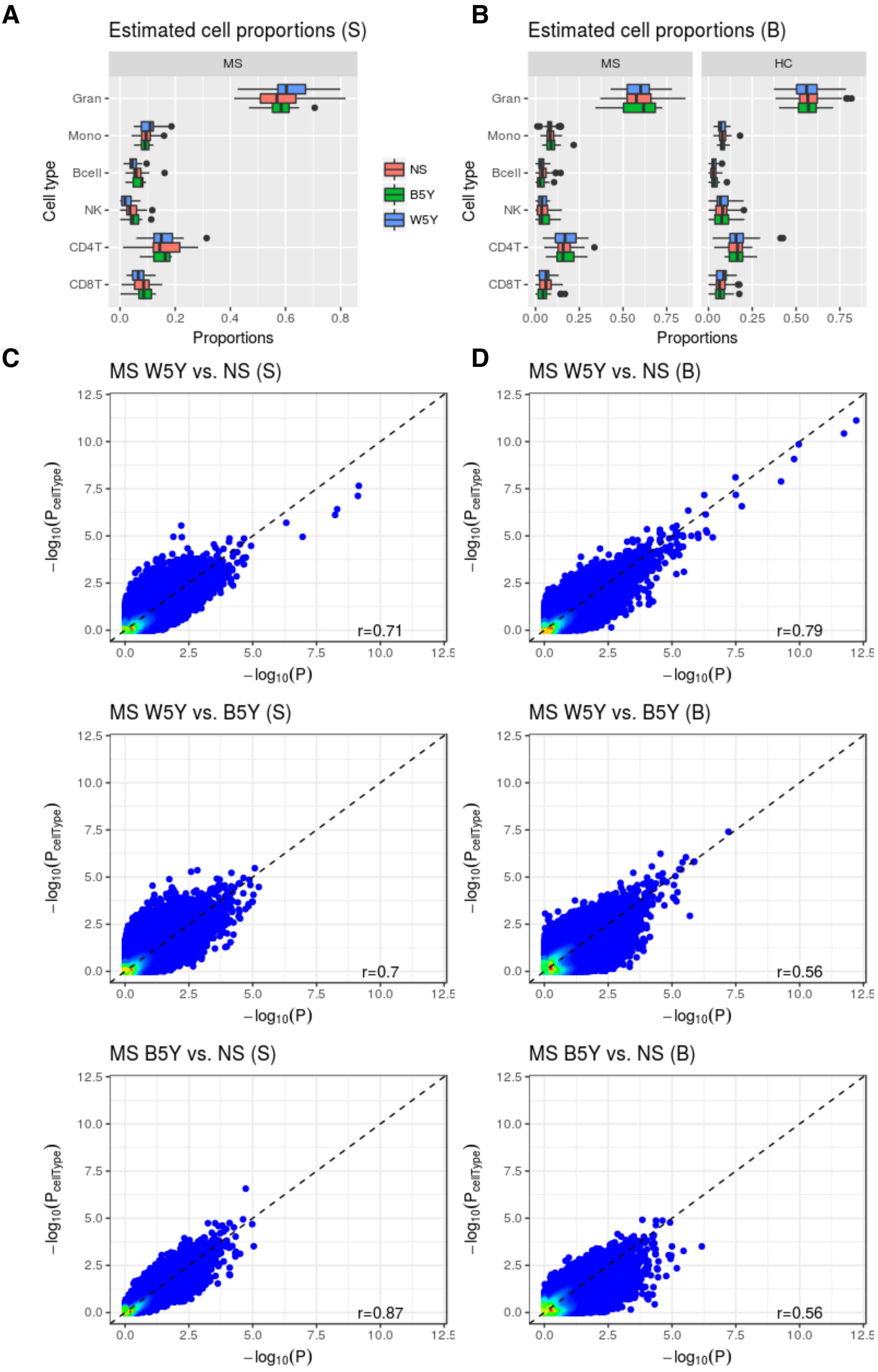

A
Estimated cell proportions (S)
MS
Gran
Mono
Bcell
NK
CD4T
CD8T
Cell type
Proportions
NS
B5Y
W5Y
B
Estimated cell proportions (B)
MS
HC
C
MS W5Y vs. NS (S)
r=0.71
D
MS W5Y vs. NS (B)
r=0.79
MS W5Y vs. B5Y (S)
r=0.7
MS W5Y vs. B5Y (B)
r=0.56
MS B5Y vs. NS (S)
r=0.87
MS B5Y vs. NS (B)
r=0.56
$-\log_{10}(P_{cellType})$
$-\log_{10}(P)$

Supp. Fig. 3

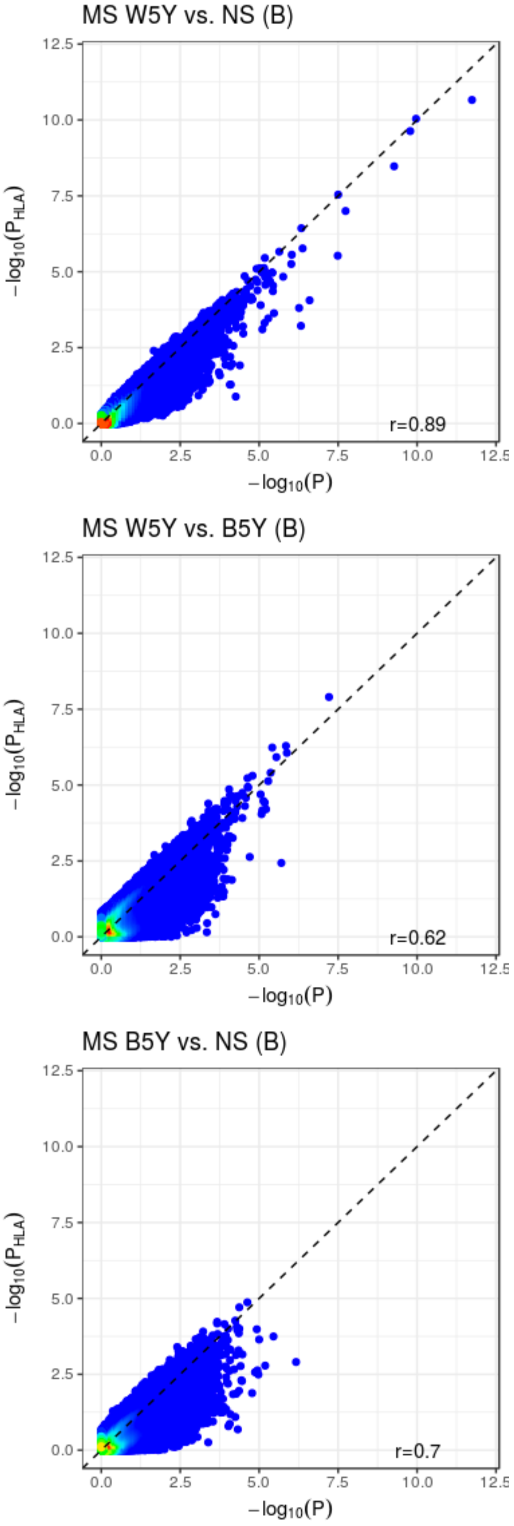
MS W5Y vs. NS (B)
r=0.89
−log10(PHLA)
−log10(P)
MS W5Y vs. B5Y (B)
r=0.62
−log10(PHLA)
−log10(P)
MS B5Y vs. NS (B)
r=0.7
−log10(PHLA)
−log10(P)

Supp. Fig. 4

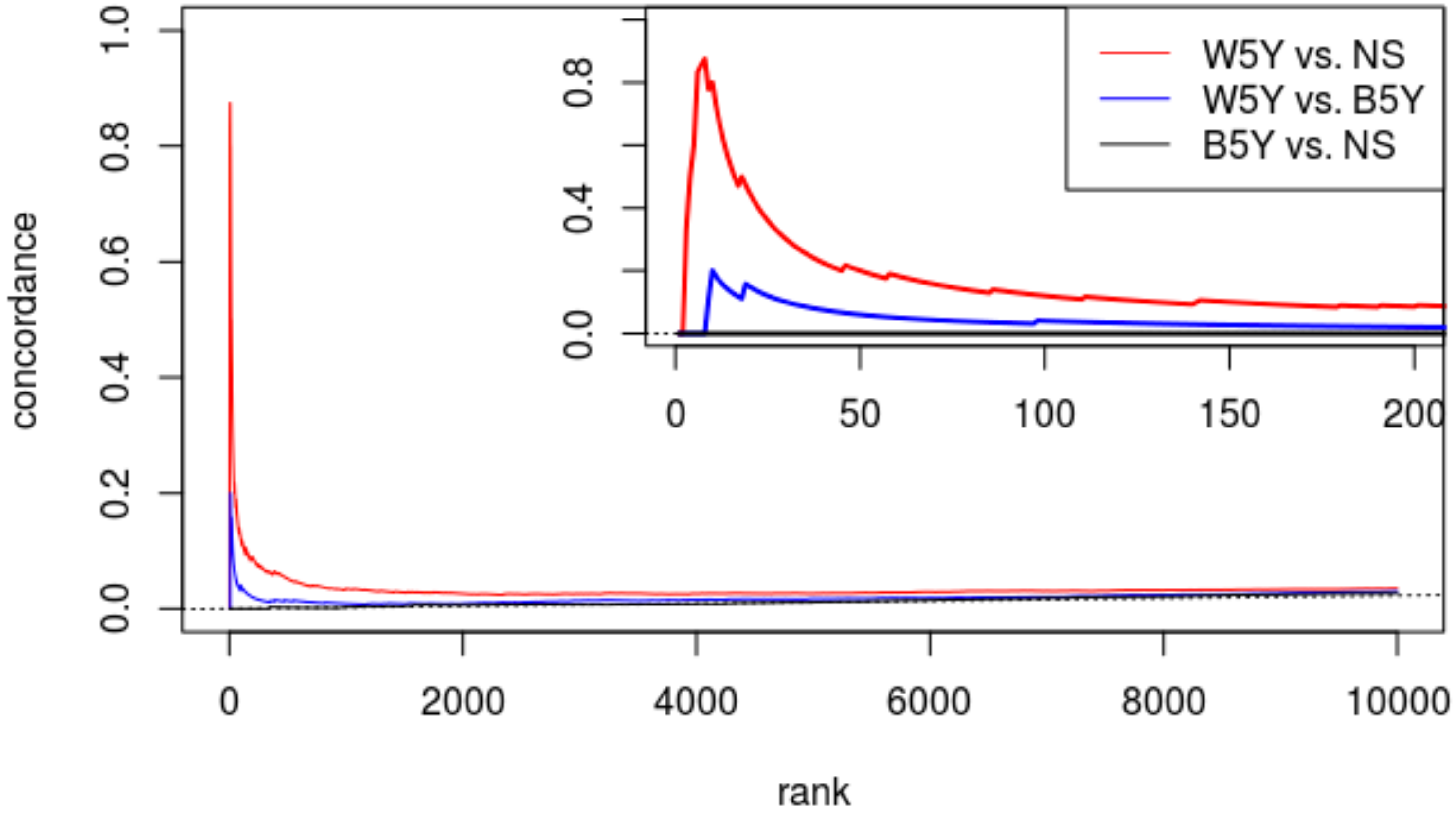

concordance
rank
0.0
0.2
0.4
0.6
0.8
1.0
0
2000
4000
6000
8000
10000
0.0
0.4
0.8
50
100
150
200
W5Y vs. NS
W5Y vs. B5Y
B5Y vs. NS

Supp. Fig. 5

S cohort

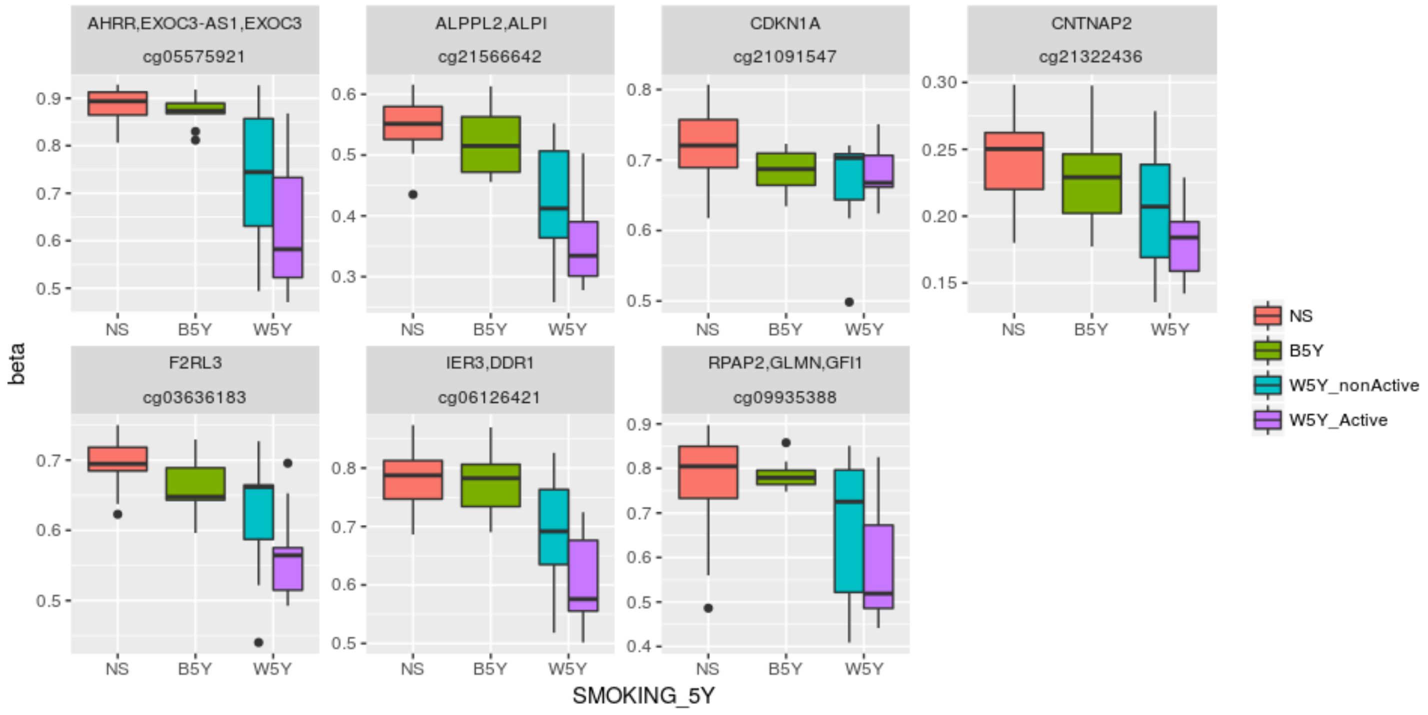

B cohort

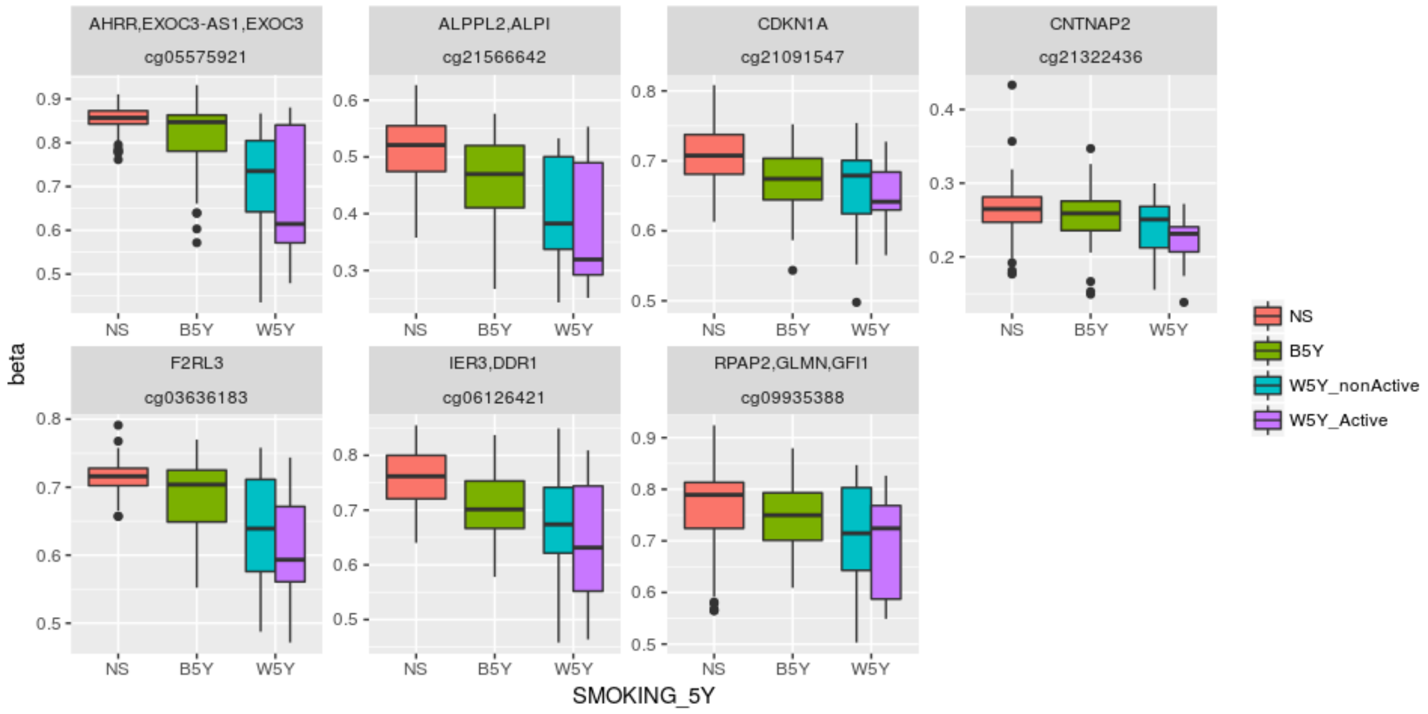

Supp. Fig. 6

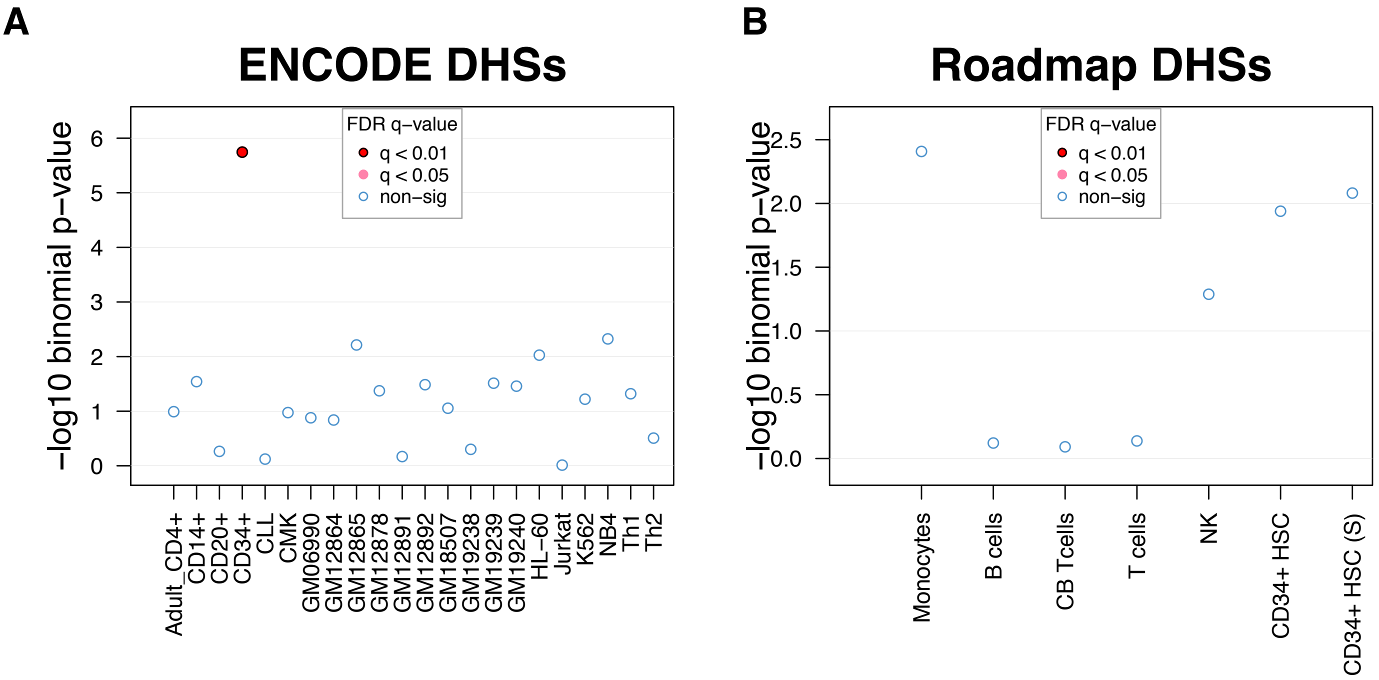
A
ENCODE DHSs
FDR q–value
q < 0.01
q < 0.05
non–sig
−log10 binomial p–value
0
1
2
3
4
5
6
Adult_CD4+
CD14+
CD20+
CD34+
CLL
CMK
GM06990
GM12864
GM12865
GM12878
GM12891
GM12892
GM18507
GM19238
GM19239
GM19240
HL–60
Jurkat
K562
NB4
Th1
Th2
B
Roadmap DHSs
FDR q–value
q < 0.01
q < 0.05
non–sig
−log10 binomial p–value
0.0
0.5
1.0
1.5
2.0
2.5
Monocytes
B cells
CB Tcells
T cells
NK
CD34+ HSC
CD34+ HSC (S)

Supp. Fig 7

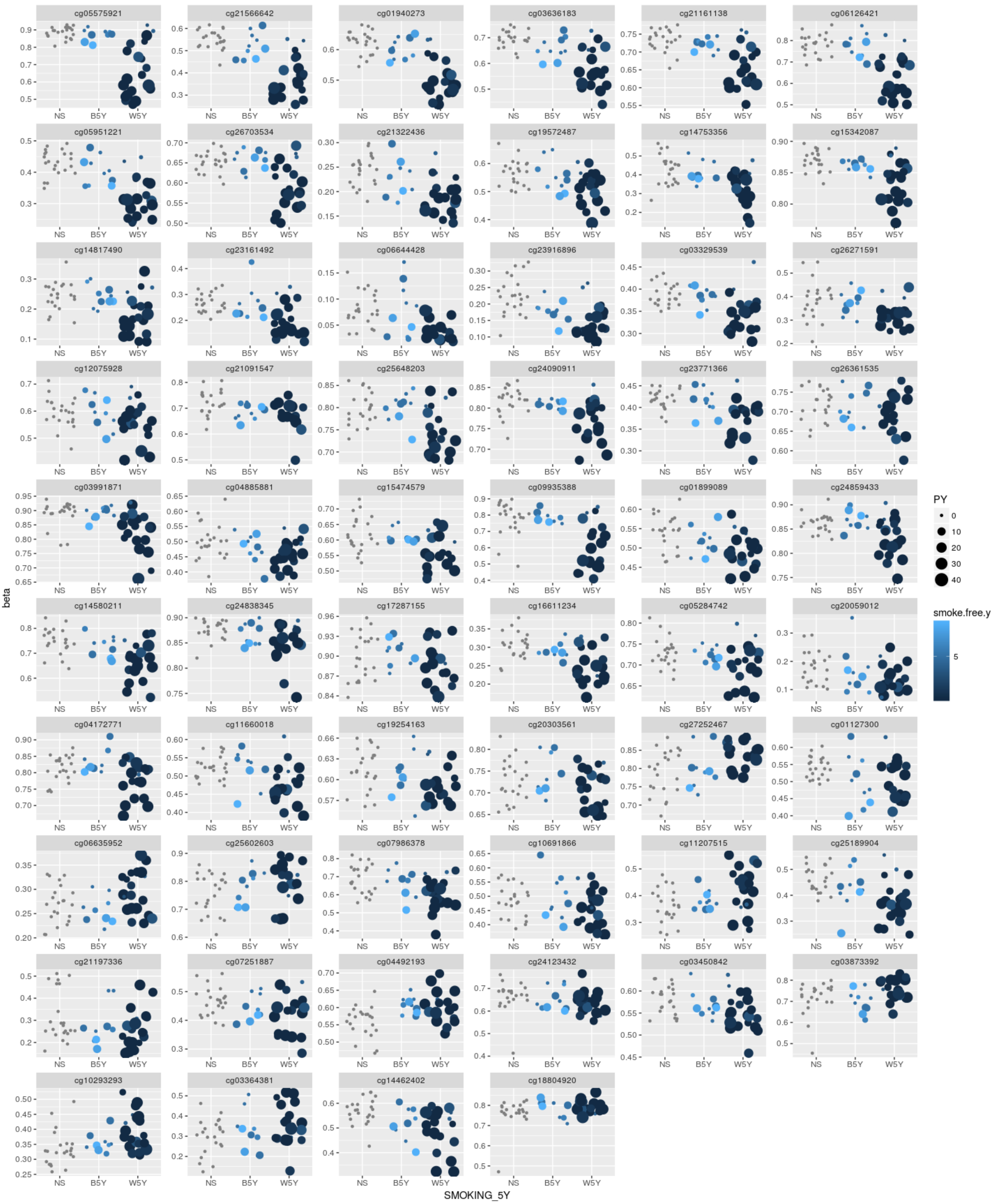
cg05575921
cg21566642
cg01940273
cg03636183
cg21161138
cg06126421
cg05951221
cg26703534
cg21322436
cg19572487
cg14753356
cg15342087
cg14817490
cg23161492
cg06644428
cg23916896
cg03329539
cg26271591
cg12075928
cg21091547
cg25648203
cg24090911
cg23771366
cg26361535
cg03991871
cg04885881
cg15474579
cg09935388
cg01899089
cg24859433
cg14580211
cg24838345
cg17287155
cg16611234
cg05284742
cg20059012
cg04172771
cg11660018
cg19254163
cg20303561
cg27252467
cg01127300
cg06635952
cg25602603
cg07986378
cg10691866
cg11207515
cg25189904
cg21197336
cg07251887
cg04492193
cg24123432
cg03450842
cg03873392
cg10293293
cg03364381
cg14462402
cg18804920
NS
B5Y
W5Y
beta
SMOKING_5Y
PY
0
10
20
30
40
smoke.free.y
5

Supp. Fig 7 (continued)

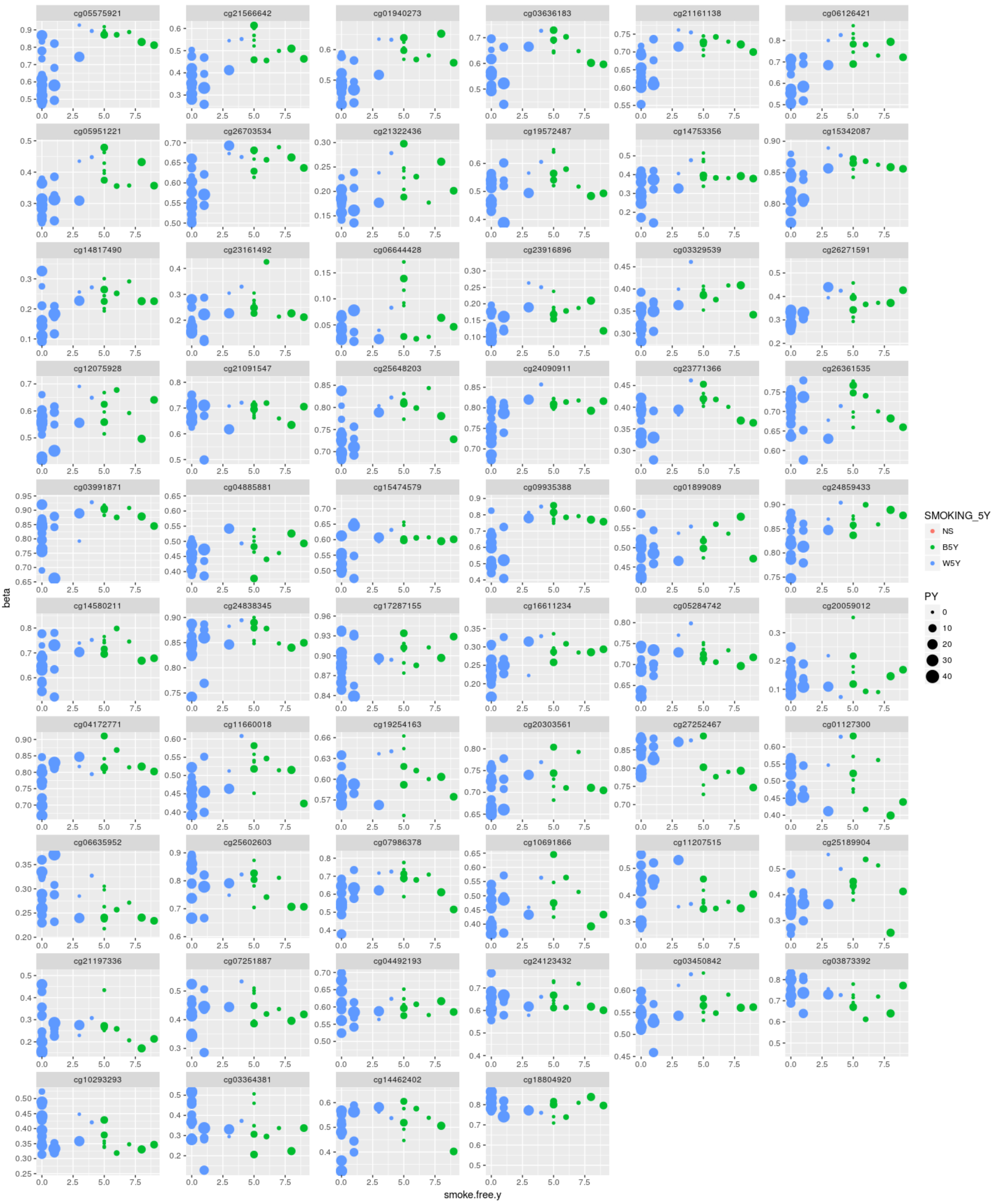

Supp. Fig. 8

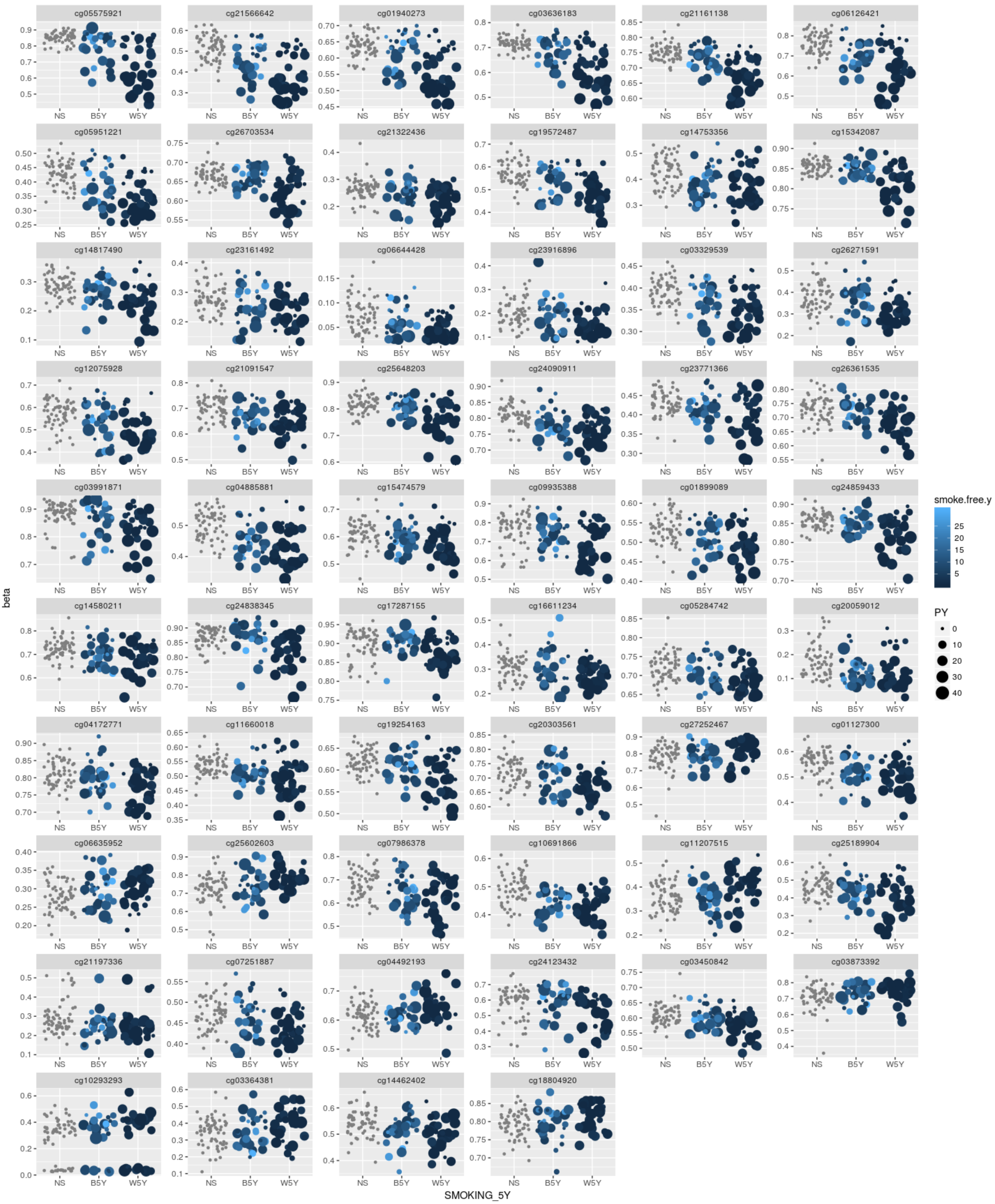

cg05575921
cg21566642
cg01940273
cg03636183
cg21161138
cg06126421
cg05951221
cg26703534
cg21322436
cg19572487
cg14753356
cg15342087
cg14817490
cg23161492
cg06644428
cg23916896
cg03329539
cg26271591
cg12075928
cg21091547
cg25648203
cg24090911
cg23771366
cg26361535
cg03991871
cg04885881
cg15474579
cg09935388
cg01899089
cg24859433
cg14580211
cg24838345
cg17287155
cg16611234
cg05284742
cg20059012
cg04172771
cg11660018
cg19254163
cg20303561
cg27252467
cg01127300
cg06635952
cg25602603
cg07986378
cg10691866
cg11207515
cg25189904
cg21197336
cg07251887
cg04492193
cg24123432
cg03450842
cg03873392
cg10293293
cg03364381
cg14462402
cg18804920
NS
B5Y
W5Y
beta
SMOKING_5Y
smoke.free.y
25
20
15
10
5
PY
0
10
20
30
40

Supp. Fig. 8 (continued)

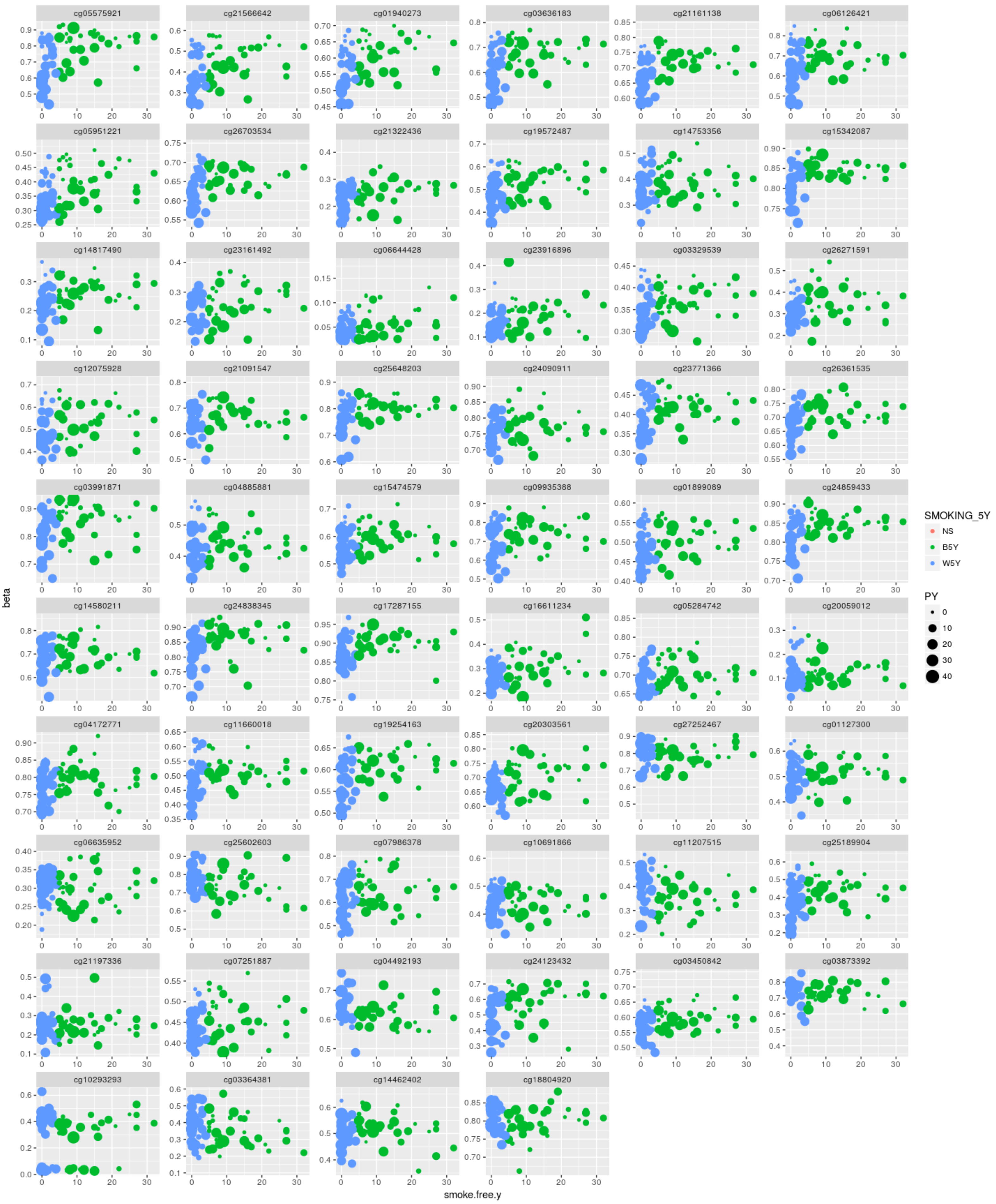

Supp. Fig. 9

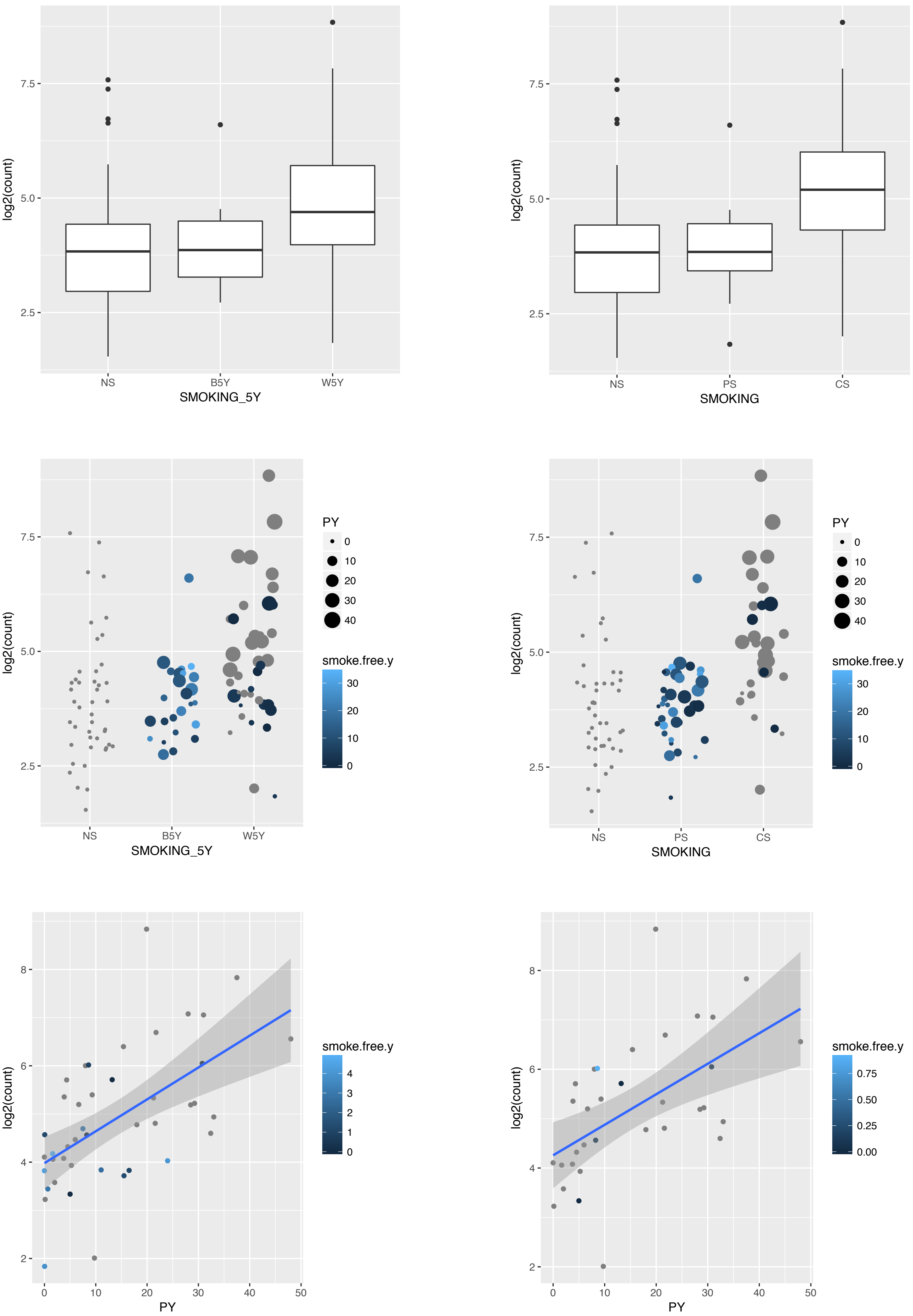
log2(count)
7.5
5.0
2.5
NS
B5Y
W5Y
SMOKING_5Y
PS
CS
SMOKING
PY
0
10
20
30
40
smoke.free.y
8
6
4
2
50
0.75
0.50
0.25
0.00

Supp. Fig. 10

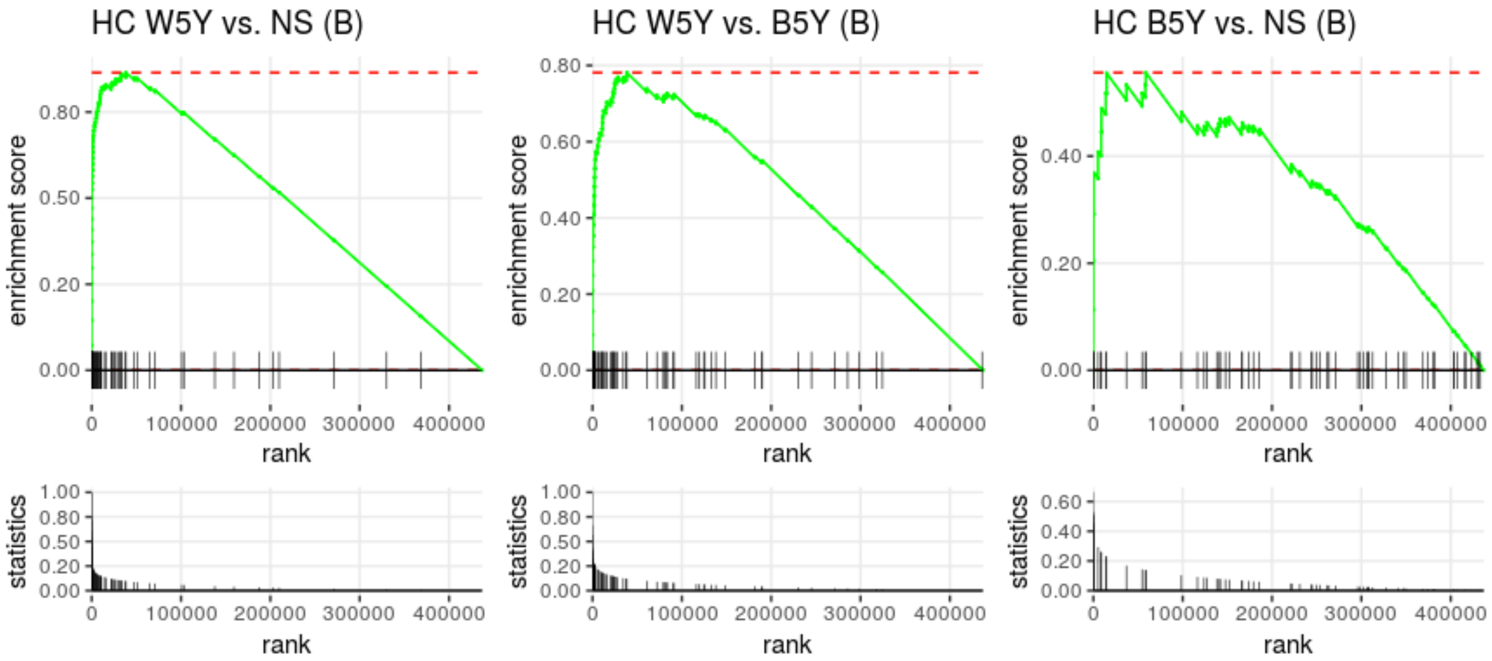
HC W5Y vs. NS (B)
HC W5Y vs. B5Y (B)
HC B5Y vs. NS (B)
enrichment score
rank
statistics
0
100000
200000
300000
400000
0.00
0.20
0.40
0.50
0.60
0.80
1.00
